# Long-term and recent trends in hypertension awareness, treatment, and control in 12 high-income countries: an analysis of 123 nationally representative surveys

**DOI:** 10.1016/S0140-6736(19)31145-6

**Published:** 2019-08-24

**Authors:** NCD Risk Factor Collaboration (NCD-RisC), Bin Zhou, Goodarz Danaei, Gretchen A Stevens, Honor Bixby, Cristina Taddei, Rodrigo M Carrillo-Larco, Bethlehem Solomon, Leanne M Riley, Mariachiara Di Cesare, Maria Laura Caminia Iurilli, Andrea Rodriguez-Martinez, Aubrianna Zhu, Kaveh Hajifathalian, Antoinette Amuzu, José R Banegas, James E Bennett, Christine Cameron, Yumi Cho, Janine Clarke, Cora L Craig, Juan J Cruz, Louise Gates, Simona Giampaoli, Edward W Gregg, Rebecca Hardy, Alison J Hayes, Nayu Ikeda, Rod T Jackson, Garry Jennings, Michel Joffres, Young-Ho Khang, Seppo Koskinen, Diana Kuh, Urho M Kujala, Tiina Laatikainen, Terho Lehtimäki, Esther Lopez-Garcia, Annamari Lundqvist, Stefania Maggi, Dianna J Magliano, Jim I Mann, Rachael M McLean, Scott B McLean, Jody C Miller, Karen Morgan, Hannelore K Neuhauser, Teemu J Niiranen, Marianna Noale, Kyungwon Oh, Luigi Palmieri, Francesco Panza, Winsome R Parnell, Markku Peltonen, Olli Raitakari, Fernando Rodríguez-Artalejo, Joel GR Roy, Veikko Salomaa, Giselle Sarganas, Jennifer Servais, Jonathan E Shaw, Kenji Shibuya, Vincenzo Solfrizzi, Bill Stavreski, Eng Joo Tan, Maria L Turley, Diego Vanuzzo, Eira Viikari-Juntura, Deepa Weerasekera, Majid Ezzati

## Abstract

**Background:**

Antihypertensive medicines are effective in reducing adverse cardiovascular events. Our aim was to compare hypertension awareness, treatment, and control, and how they have changed over time, in high-income countries.

**Methods:**

We used data from people aged 40–79 years who participated in 123 national health examination surveys from 1976 to 2017 in 12 high-income countries: Australia, Canada, Finland, Germany, Ireland, Italy, Japan, New Zealand, South Korea, Spain, the UK, and the USA. We calculated the proportion of participants with hypertension, which was defined as systolic blood pressure of 140 mm Hg or more, or diastolic blood pressure of 90 mm Hg or more, or being on pharmacological treatment for hypertension, who were aware of their condition, who were treated, and whose hypertension was controlled (ie, lower than 140/90 mm Hg).

**Findings:**

Data from 526 336 participants were used in these analyses. In their most recent surveys, Canada, South Korea, Australia, and the UK had the lowest prevalence of hypertension, and Finland the highest. In the 1980s and early 1990s, treatment rates were at most 40% and control rates were less than 25% in most countries and age and sex groups. Over the time period assessed, hypertension awareness and treatment increased and control rate improved in all 12 countries, with South Korea and Germany experiencing the largest improvements. Most of the observed increase occurred in the 1990s and early-mid 2000s, having plateaued since in most countries. In their most recent surveys, Canada, Germany, South Korea, and the USA had the highest rates of awareness, treatment, and control, whereas Finland, Ireland, Japan, and Spain had the lowest. Even in the best performing countries, treatment coverage was at most 80% and control rates were less than 70%.

**Interpretation:**

Hypertension awareness, treatment, and control have improved substantially in high-income countries since the 1980s and 1990s. However, control rates have plateaued in the past decade, at levels lower than those in high-quality hypertension programmes. There is substantial variation across countries in the rates of hypertension awareness, treatment, and control.

**Funding:**

Wellcome Trust and WHO.

## Introduction

High blood pressure is one of the most important risk factors for stroke, heart disease, and kidney disease.^1^ Antihypertensive medicines can effectively reduce blood pressure and the risk of associated diseases.^2^, ^3^ As clinical trials have shown the benefits of pharmacological treatment for patients with low to moderate blood pressure, clinical guidelines have evolved to recommend lower blood pressure thresholds for initiating treatment. National and regional hypertension programmes (eg, the Canadian Hypertension Education Program and Kaiser Permanente Northern California hypertension programme^4^, ^5^) have demonstrated that it is feasible to achieve a high level of hypertension control by improving health-care provider and patient compliance with evidence-based guidelines, establishing a hypertension registry, monitoring physician performance and providing feedback, and implementing regular blood pressure measurements and single-pill combination therapy.^4^

There are, however, few data on how different high-income countries, with different health systems and clinical guidelines, compare in terms of hypertension awareness, treatment, and control; how comparative performance in these countries has changed over time; and which countries need to improve hypertension management. We aimed to benchmark hypertension awareness, treatment, and control across 12 high-income countries over a period of nearly four decades using national data.

## Methods

### Data sources

In this analysis, we used data from 123 national health examination surveys that were done from 1976 to 2017 in 12 high-income countries: Australia, Canada, Finland, Germany, Ireland, Italy, Japan, New Zealand, South Korea, Spain, the UK, and the USA. These surveys measured blood pressure in random samples of the general population. A list of surveys used and information about their study designs, including age range and number of participants, number of blood pressure measurements taken, cuff size, and type of device used to measure blood pressure, are provided in the appendix (pp 3–6).

**Research in context**

**Evidence before this study**

We searched MEDLINE (via PubMed) for articles published from inception to Jan 15, 2019, using the search terms ((hypertension[Title] AND (((medication OR treatment) AND control) OR aware*) AND “blood pressure”) OR (cardiovascular[Title] AND risk factor*[Title] AND “blood pressure” AND (((medication OR treatment) AND control) OR aware*))) AND (trend* OR global OR worldwide) NOT patient*[Title]. No language restrictions were applied. We found some studies on trends in hypertension prevalence, awareness, treatment, and control in individual countries. Only three of these studies, in the USA and South Korea, used post-2010 data and reported a plateau in hypertension treatment and control. We found a few studies or reviews that compared hypertension awareness, treatment, and control across countries at one point in time, mostly using data collected from the 1980s to the 2000s. These studies mostly compared high-income countries as a group with low-income and middle-income countries, and they did not assess change over time. One study reported change in hypertension prevalence, awareness, treatment, and control in 21 countries using subnational data from the MONICA Project between two points in time (late 1980s and early 1990s). Another study, a systematic review of published studies, reported hypertension prevalence, awareness, treatment, and control in two points in time (2000 and 2010). Both of these studies did not examine trends over time in detail and did not use data collected after 2010. To our knowledge, there are no comparative studies of long-term and recent trends in hypertension awareness, treatment, and control in high-income countries.

**Added value of this study**

This study provides the most comprehensive analysis of trends in hypertension awareness, treatment, and control in high-income countries using national surveys. By covering a substantially longer time period than previous studies, we noted not only substantial improvements in hypertension awareness, treatment, and control since hypertension treatment was incorporated in clinical guidelines, but also variations in the uptake of treatment and success of control across countries. By use of up-to-date national surveys in each country, we could evaluate recent trends, which revealed a plateau of the improvements in hypertension treatment coverage and control in most countries.

**Implications of all the available evidence**

There has been substantial improvement in hypertension awareness, treatment, and control in high-income countries since the 1980s and 1990s, most of which was achieved in the late 1990s and early 2000s. Canada, Germany, South Korea, and the USA have the highest rates of awareness, treatment, and control, whereas Finland, Ireland, Japan, and Spain have the lowest. Even in the best performing countries, the rates fall short of those achieved in high-quality hypertension programmes—eg, the Kaiser Permanente Northern California hypertension programme. There is need for strategies that further improve the diagnosis, treatment, and control of hypertension in high-income countries.

We used data on men and women aged 40–79 years. We did not use data on participants younger than 40 years because hypertension is less common in these ages. We did not use data on participants older than 80 years because guidelines recommend different treatment pathways and goals in older ages.

### Data analysis

All analyses were done by sex and 10-year age groups. In surveys that covered only a part of the 10-year age group, we used the data only when the age range was 5 years or more; this criterion led to exclusion of data on 220 participants (<0·1%). In each survey, we identified the question used to establish whether a participant had been diagnosed with hypertension, often worded as a variation of: “have you ever been told by a doctor or other health professional that you had hypertension, also called high blood pressure?” We did not consider having had hypertension diagnosis only during pregnancy as a previous diagnosis. We established whether participants had been pharmacologically treated for hypertension using survey-specific questions, which were typically worded in the following way: “Because of your hypertension, have you ever been told to take prescribed medicine? Are you now taking it?” “Are you currently taking any medicines, tablets or pills for high blood pressure?” We took a similar approach in surveys that had gathered information on medicines prescribed to the participants, by relying on survey information about hypertension being the purpose or diagnosis leading to taking a blood-pressure-lowering medicine. Participants with missing data on blood pressure, diagnosis, or treatment were excluded from the analysis (2% of data). After exclusion, we had data on 526 336 participants.

In each survey, we calculated the prevalence, and its 95% CI, of hypertension, which was defined as systolic blood pressure of 140 mm Hg or more, or diastolic blood pressure of 90 mm Hg or more, or being on pharmacological treatment for hypertension. We also calculated the proportion of participants with hypertension who reported having been diagnosed (awareness), who were using medication to treat hypertension (treatment), and who had systolic blood pressure of less than 140 mm Hg and diastolic blood pressure of less than 90 mm Hg (control). Blood pressure of each participant was measured more than once in all surveys, except those in Japan before 2000. When more than one measurement was taken, we discarded the first and used the remainder for the aforementioned analyses, by averaging when more than two measurements were taken. 37 surveys with 175 437 participants had data on hypertension treatment but not on diagnosis. 27 of these surveys were from Japan, which in its annual national survey asks questions about treatment every year but about diagnosis every 10 years. For these surveys, we calculated hypertension prevalence, treatment, and control, but not awareness. Where relevant, we accounted for complex survey design and used survey sample weights when calculating prevalence, awareness, treatment, and control.

Immediate pharmacological treatment is recommended for people with stage 2 hypertension (ie, systolic blood pressure of 160 mm Hg or more, or diastolic blood pressure of 100 mm Hg or more) by all hypertension guidelines, and failure to provide such treatment is a shortcoming in the health-care system. Therefore, we also calculated the proportion of people with hypertension who had stage 2 hypertension but were unaware of or untreated for their condition.

### Role of the funding source

The funder of the study had no role in study design, data collection, analysis, interpretation, or writing of the report. BZ had full access to the data in the study. The corresponding author had final responsibility for the decision to submit for publication.

## Results

**Figure 1:**
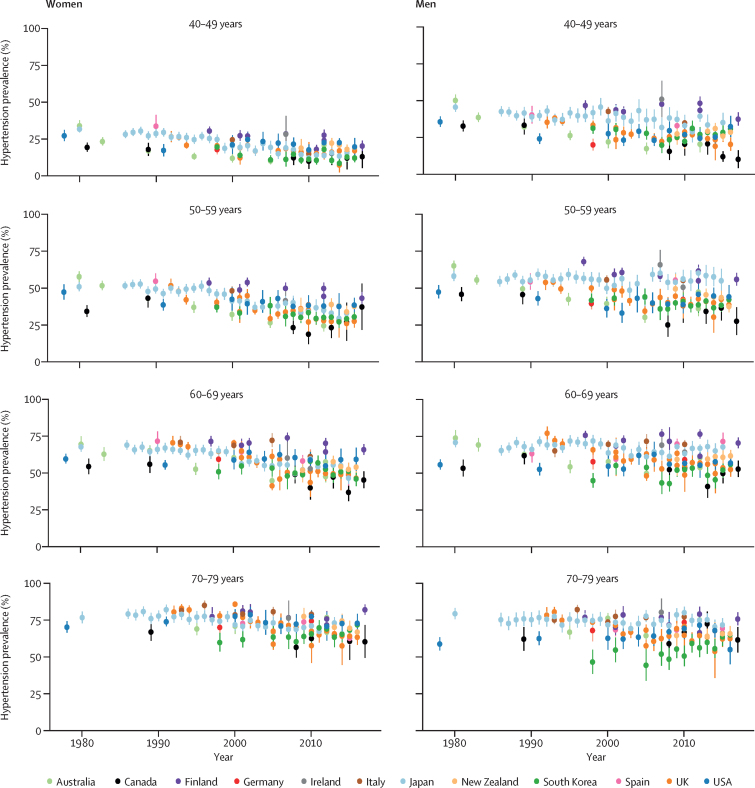
Trends in hypertension prevalence by country, sex, and age group See appendix (pp 29–41) for country-by-country results. Error bars indicate 95% CIs.

**Figure 2:**
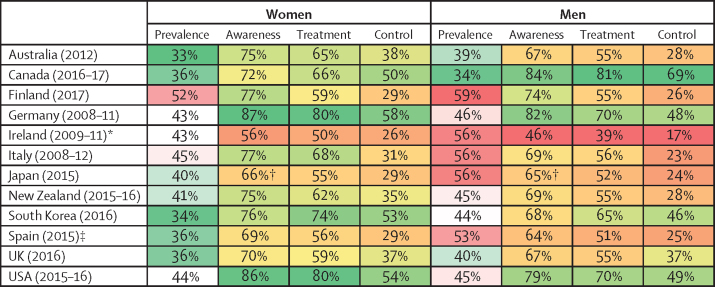
Prevalence of hypertension and rates of awareness, treatment, and control in women and men aged 40–79 years Data are from the latest national survey in each country. Results shown are crude (ie, not age-standardised) to reflect the total burden of hypertension and its awareness, treatment, and control. Age-specific results, and their uncertainty, are available in Figure 1, Figure 3, Figure 4, Figure 5, Figure 6, and the appendix (pp 7–9). For each outcome, the colour range for cells extends from lowest to highest value. Men and women share the same colour scheme. Awareness, treatment, and control are reported as the proportions. *The latest national survey in Ireland had data for people aged 50 to 79 years; data from an earlier survey in 2007 were used for people aged 40 to 49 years. †The question on awareness was not asked in 2015 in Japan; awareness data from 2010 were used. ‡The latest national survey in Spain had data for people aged 60 to 79 years; data from an earlier survey in 2009 were used for people aged 40 to 59 years.

In the most recent national surveys, Canada, South Korea, Australia, and the UK had the lowest prevalence of hypertension, and Finland the highest (Figure 1, Figure 2). Prevalence was also higher than 50% in men in Ireland, Italy, Japan, and Spain. In ages 40–49 years, hypertension prevalence ranged from 12% (95% CI 9–15; South Korea) to 20% (17–24; Finland) in women, and from 10% (4–16; Canada) to 37% (33–42; Finland) in men. In ages 70–79 years, prevalence ranged from 61% (49–72; Canada) to 82% (79–86; Finland) in women, and from 55% (45–65; USA) to 77% (74–80; Italy) in men (figure 1; appendix pp 7–9). Overall prevalence across all participants aged 40–79 years ranged from 33% in Australia to 52% in Finland in women, and from 34% in Canada to 59% in Finland in men (figure 2).

In most countries and among age and sex groups, hypertension prevalence did not change over time, although some age and sex groups showed a decline, especially after the mid-2000s (figure 1; see appendix p 10 for p values for change since 2005). The only group in which hypertension prevalence increased was South Korean men and women aged 70–79 years.

**Figure 3:**
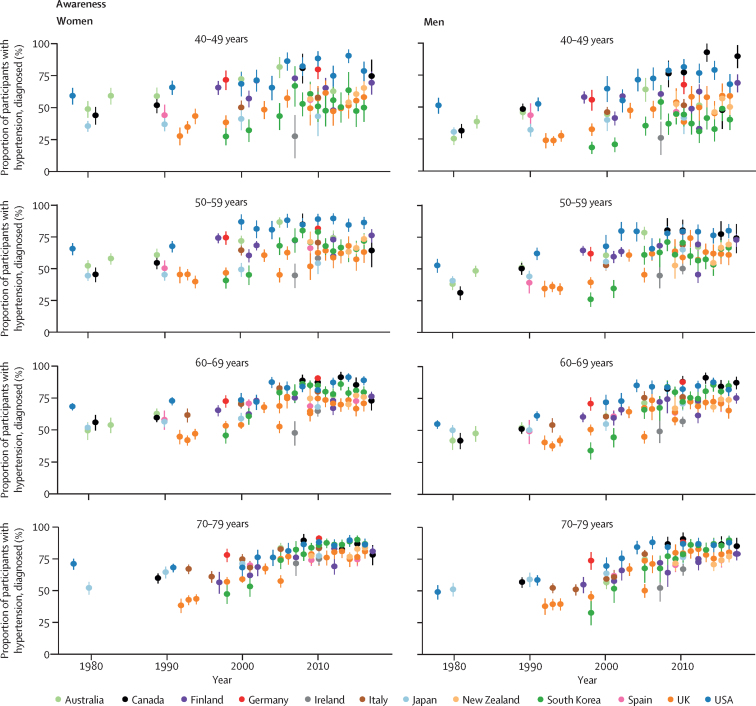

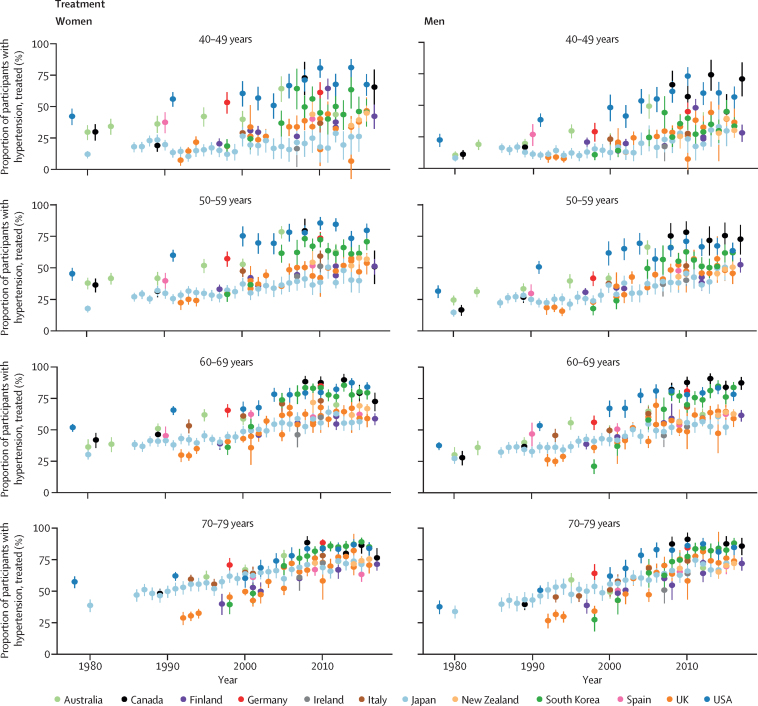
Trends in hypertension awareness and treatment among people with hypertension, by country, sex, and age group See appendix (pp 29–41) for country-by-country results. Error bars indicate 95% CIs.

**Figure 4:**
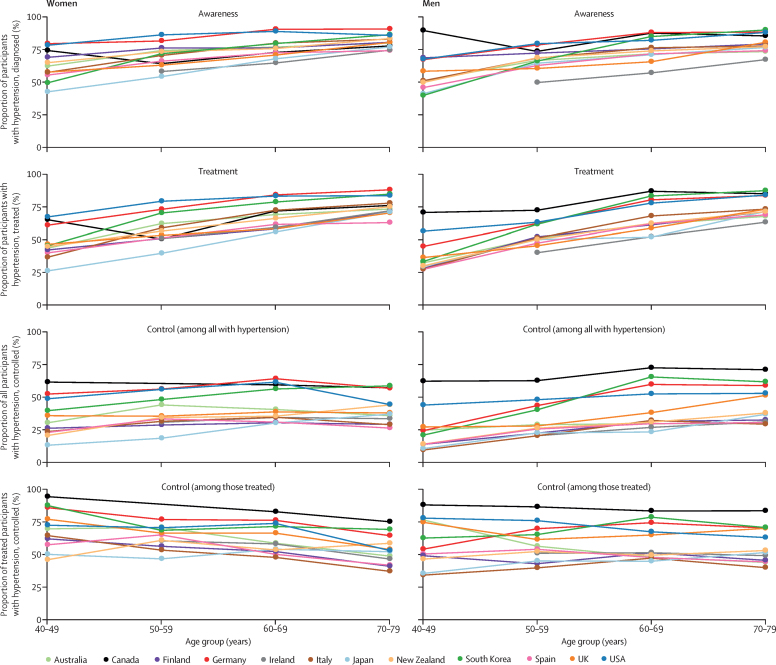
Age patterns of hypertension awareness, treatment, and control among women and men, according to the latest national surveys

Awareness and treatment of hypertension increased in most countries (figure 3). Much of the improvement happened before the mid-2000s, and awareness and treatment have plateaued since, at levels between 40% and 80% depending on age (figure 3; see appendix pp 11–12 for p values for change since 2005). Awareness and treatment rates were lower in younger age groups than in older ones (figure 4).

The USA started with higher rates of awareness (about 50% or more in different age groups) and treatment (about 25% or more in different age groups) than other countries in the 1980s and 1990s, and has largely maintained this advantage (Figure 2, Figure 3). In their most recent surveys, Canada, Germany, and South Korea also had high rates of hypertension awareness and treatment, in some age and sex groups having surpassed the USA. The USA, Canada, and Germany not only had high awareness and treatment rates, but also a smaller age gradient in awareness and treatment (figure 4), meaning that diagnosis and treatment are benefiting all ages. Australia, which had similar rates of awareness and treatment to the USA in the 1990s, has fallen behind, with awareness and treatment for men now in the lower half of these 12 countries (figure 2). By contrast, South Korea and the UK started with relatively low rates of hypertension awareness and treatment, but have closed the gap with (the UK) or outperformed (South Korea) most other countries because of a sharp increase in the late 1990s and early 2000s. There might, however, have been a slight decrease in hypertension awareness and treatment in the UK and in Canadian women in the past 5 years (appendix pp 31, 40), which needs to be confirmed when additional years of data become available. Finally, although hypertension awareness and treatment increased in Japan at a steady pace since the 1980s, it remains lower than in most other countries.

**Figure 5:**
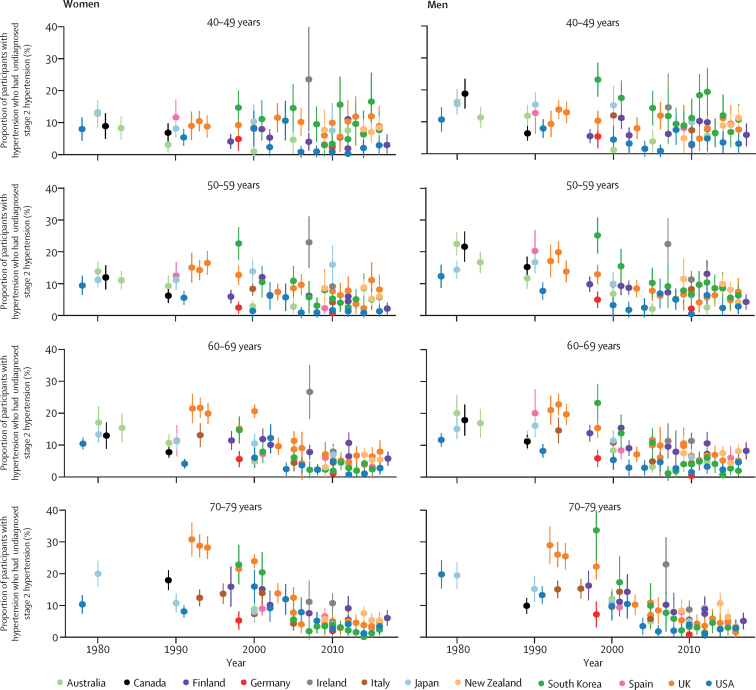

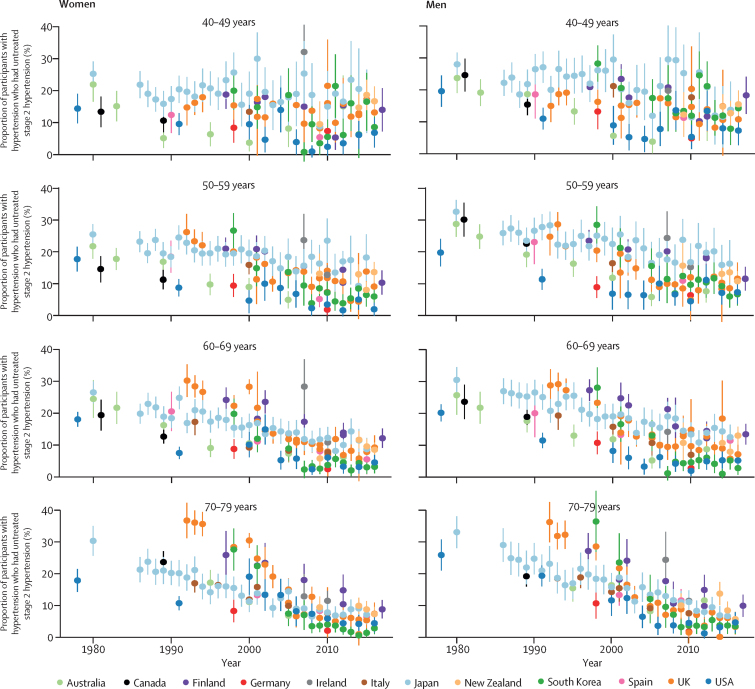
Trends in proportion of people with hypertension who had undiagnosed or untreated stage 2 hypertension, by country, sex, and age group Error bars indicate 95% CIs.

As awareness and treatment rates increased, the proportion of people with hypertension who had stage 2 hypertension but were not diagnosed or treated declined markedly, especially in older ages (figure 5). In the latest surveys, this proportion was less than 5% in most age groups in Germany and the USA and in older age groups in South Korea. By contrast, in some age and sex groups in Finland, Italy, Japan, and New Zealand, 15–25% of participants with hypertension had untreated stage 2 hypertension.

**Figure 6:**
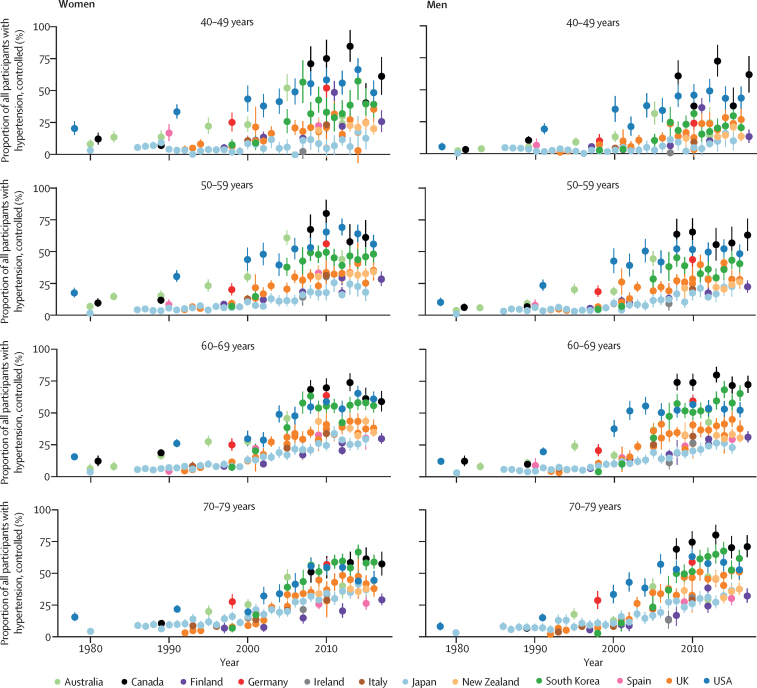

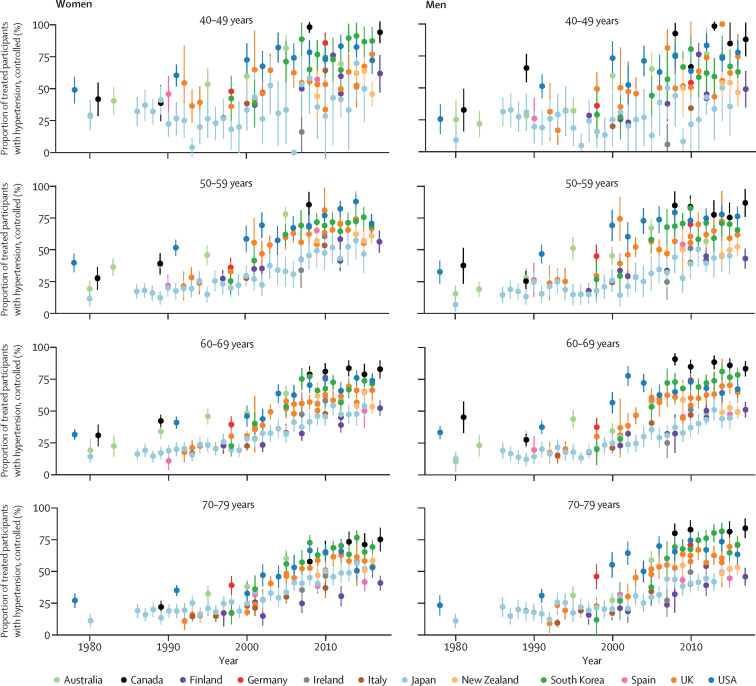
Trends in hypertension control, by country, sex, and age group See appendix (pp 29–41) for country-by-country results. Error bars indicate 95% CIs.

In the 1980s and early 1990s, the proportion of people with hypertension whose hypertension was controlled (ie, had achieved systolic blood pressure of less than 140 mm Hg and diastolic blood pressure of less than 90 mm Hg) was less than 25% in most countries and age and sex groups (figure 6). Control rates improved over time, reaching 60–70% in some countries and age and sex groups. Like awareness and treatment rates, this increase mostly happened in the 1990s and early-mid 2000s, with slower improvement since then followed by a plateau after the mid-2000s in most countries and age groups (see appendix p 13 for p values for change since 2005). Even in countries with the highest rates of control—namely, Canada, South Korea, the USA, and Germany—the plateau occurred below 70% when taken across the entire 40–79-year age range (figure 2). Hypertension control was lower in Finland, Ireland, Italy, Japan, and Spain than in other countries, with control rates being less than 20% in some age and sex groups. Taken across the entire 40–79 years, control rates in Canada and Germany (50–58% for women and 48–69% for men) were two to four times those in Ireland, Japan, Italy, Spain, and Finland (26–31% for women and 17–26% for men). Suboptimal control was partly because some people with hypertension were not treated, and partly because control rate among those treated was at most around 80% (figure 6).

## Discussion

By use of data on more than 520 000 participants in 123 national health examination surveys from 12 high-income countries, we found that hypertension awareness, treatment, and control have improved substantially in all 12 countries since the 1980s and 1990s. However, control rates have plateaued in the past decade, at levels lower than those in high-quality hypertension programmes, such as the Kaiser Permanente Northern California hypertension programme.^4^ Suboptimal control occurs partly because some people with hypertension, even with stage 2 hypertension, are not diagnosed or treated, and partly because at least 20% of those diagnosed and treated fail to achieve control. There were nonetheless variations across countries in both hypertension prevalence and how health systems detect and treat hypertension. Although the USA consistently performed better than most of its comparators in terms of awareness, treatment, and control, Canada, Germany, and South Korea have achieved substantial improvements and now perform as well as, or better than, the USA. Finland, Japan, Ireland, Italy, Spain, and men in New Zealand had lower rates of awareness, treatment, and control than most other countries assessed.

To our knowledge, there are no multinational studies comparing trends in hypertension awareness, treatment, and control in high-income countries. A few cross-sectional studies or reviews compared prevalence, awareness, treatment, and control using national or subnational data mostly between the 1980s and 2000s.^6^, ^7^, ^8^, ^9^, ^10^, ^11^, ^12^, ^13^, ^14^ These studies mostly compared high-income countries as a group with low-income and middle-income countries and did not examine variations among high-income countries. Change over time has been reported primarily in individual countries, with the exception of the MONICA study,^7^ which reported change from the mid-1980s to the mid-1990s using subnational data, and a systematic review of published studies^9^ that reported on hypertension for two points in time (2000 and 2010). These studies did not examine trends over time in detail, including the rapid increase in diagnosis and treatment in the late 1990s and early 2000s, and they did not find the recent plateau as we reported here because they did not have data after 2010. The findings of these studies on countries with better (eg, Canada and the USA) and worse (eg, the UK) performance were largely consistent with ours.^7^, ^8^, ^9^, ^10^, ^11^ Only two studies from the USA^15^, ^16^ and one from South Korea^17^ reported a plateau in hypertension treatment and control, as we reported here, because previous studies used fewer and older data sources, largely before 2010. Given the large variation across these 12 countries in the rates of awareness, treatment, and control, understanding health system performance in hypertension treatment in other high-income countries requires local data.

The strengths of our study include its scope of comparative analysis in 12 countries over around four decades, which allowed trends, including accelerations and plateau, and variations across countries, to be uncovered, and the large number of high-quality national surveys used in the analysis. A limitation of data was that fewer surveys were available before 1990, which restricted comparisons in the early part of the analysis period. Some surveys did not have data on hypertension diagnosis; nonetheless, countries with such features (eg, Japan) still had sufficient data to evaluate trends in awareness. Furthermore, survey protocols might differ across countries because of differences in their geographical and social circumstances. For example, the time before blood pressure is measured might differ across surveys. Nonetheless, surveys commonly start with an interview module before moving to physical measurements, which allows sufficient time for stabilisation of blood pressure, and all survey protocols include a resting time before blood pressure measurement.^18^ Finally, over time, standard mercury sphygmomanometers have been replaced by random-zero sphygmomanometers and, more recently, digital oscillometric devices in some health surveys. Surveys used in our study used the same type of device over time in each country, except those in Australia, Canada, Germany, and Spain, where the measurement instrument used changed over time (appendix pp 4–6). The effect of measurement device on hypertension prevalence depends on the circumstances of each survey. For example, an automated digital device, although not the traditional gold standard in a clinical setting, might help to reduce potential observer bias, compared with a standard mercury sphygmomanometer.^19^ However, measurements from different devices might not be fully comparable.^20^

The increase in hypertension awareness, treatment, and control, especially in the 1990s and early 2000s, was probably due to more widespread uptake of, and compliance with, clinical guidelines for hypertension with simplified recommendations (see appendix pp 14–24 for details on national and international guidelines). The progressively lower thresholds to diagnose hypertension and initiate treatment also contributed to higher rates of awareness, treatment, and control based on the threshold of 140/90 mm Hg. Over time, newer drugs became available (eg, renin–angiotensin system inhibitors and calcium-channel blockers),^21^ and improved treatment efficacy and control for some patients and had smaller side-effects than did the older generation ones (eg, thiazide diuretics).^22^ Because multiple agents are usually needed to achieve blood pressure targets, the use of fixed-dose combination therapy is likely to have reduced medication regimen complexity and improved treatment adherence and control.^23^ Nationally implemented screening and health check-up programmes (eg, in Canada^24^ and South Korea^25^), and tighter control targets might also have contributed to the observed improvements.

Low rates of awareness, treatment, and control in some countries might be partly related to higher thresholds for pharmacological treatment in clinical guidelines for hypertension. For example, in Finland, which had one of the lowest rates of treatment and control, the threshold for treatment was only recently lowered to 140/90 mm Hg (appendix p 17).^26^ Guidelines also differ in their recommendations for patients with blood pressure between 140/90 mm Hg and 160/100 mm Hg and with low levels of other risk factors (appendix pp 14–24). Some recommend immediate treatment,^24^, ^27^ others recommend starting with lifestyle changes before initiating treatment,^28^, ^29^ and yet others do not make an explicit recommendation for this group.^30^, ^31^, ^32^ As a result, physicians in some countries are less likely to treat patients with blood pressure between 140/90 mm Hg and 160/100 mm Hg than in other countries—eg, Japan compared with the USA.^33^, ^34^ The extent of reliance on total cardiovascular risk versus blood pressure might also contribute to the observed variation in treatment across countries. In particular, New Zealand, which had below average treatment coverage in our study, has stopped publishing dedicated clinical guidelines for hypertension and relies on total cardiovascular risk for treatment decisions except for stage 2 hypertension.^32^ Beyond guidelines, health system characteristics that facilitate or impede contact with providers, including insurance, co-payment for physician consultation or prescription, and pay-for-performance incentives to physicians, might affect how often people see their physicians and have their blood pressure measured, whether antihypertensive medicines are prescribed, and whether patients comply with the treatment (see appendix pp 25–28 for information on health systems in these 12 countries). In addition to these systems determinants, the countries with the best hypertension control—ie, Canada, the USA, South Korea, and Germany—all have national programmes for hypertension education or health check-up (appendix pp 25–28). Detailed evaluation of the Canadian Hypertension Education Program shows that annual update of recommendations, tailored knowledge dissemination, coordinated implementation and outcome evaluation, and national leadership are also important for the success of national hypertension programmes.^35^

Randomised trials over half a century have shown the benefits of treating hypertension, at progressively lower thresholds and with multiple drugs. Our results show that when incorporated in guidelines and implemented in effective health systems, treatment coverage and control rates can rapidly increase. Yet even in these well resourced countries, treatment coverage and control vary substantially across countries, and rarely reach the levels achieved in high-quality regional hypertension programmes. The increasing use of fixed-dose combination therapy can help to increase control rates among treated patients.^36^ Increasing diagnosis and treatment, however, requires mechanisms and incentives that increase contact with health-care systems, especially for disadvantaged groups that typically have higher prevalence of hypertension and lower rates of diagnosis and treatment.^37^, ^38^ National health systems should set ambitious targets, and experiment and rigorously evaluate innovative mechanisms at the facility and community levels^4^, ^38^, ^39^ to improve hypertension awareness, treatment, and control. Doing so would help to address the large burden of uncontrolled hypertension.^40^
