# Supplementary material for: Long-term and recent trends in hypertension awareness, treatment, and control in 12 high-income countries: an analysis of 123 nationally representative surveys
Source: Lancet. 2019 Aug 24;394(10199):639–51. doi: 10.1016/S0140-6736(19)31145-6 (PMC6717084; doi:10.1016/S0140-6736(19)31145-6)
Supplement: Supplementary appendix [file mmc1.pdf]

THE LANCET

Supplementary appendix

This appendix formed part of the original submission and has been peer reviewed. We post it as supplied by the authors.

Supplement to: NCD Risk Factor Collaboration (NCD-RisC). Long-term and recent trends in hypertension awareness, treatment, and control in 12 high-income countries: an analysis of 123 nationally representative surveys. *Lancet* 2019; published online July 18. http://dx.doi.org/10.1016/S0140-6736(19)31145-6.

Appendix

Long-term and recent trends in hypertension awareness, treatment, and control in 12 high-income countries: an analysis of 123 nationally representative surveys

NCD Risk Factor Collaboration (NCD-RisC)

Appendix Table 1. Data availability by country.

Country	Start year	End year	Number of data sources (number of data sources with diagnosis information)
Australia	1980	2012	8 (6)
Canada	1981	2017	7 (7)
Finland	1997	2017	9 (9)
Germany	1998	2011	2 (2)
Ireland	2007	2010	2 (2)
Italy	1992	2012	6 (6)
Japan	1980	2015	31 (4)
New Zealand	2009	2016	5 (4)
South Korea	1998	2016	13 (13)
Spain	1990	2015	4 (4)
United Kingdom	1992	2016	25 (18)
United States of America	1976	2016	11 (11)
**Total**	**1976**	**2017**	**123 (86)**

Appendix Table 2. Data sources used in the analysis.

	Country	Data years	Survey/Study name/Citation	Age range as in the NCD-RisC database		Sample size		Number of blood pressure measurements taken	Whether multiple cuff sizes were available for measurement	Type of device used to measure blood pressure	Note
				Male	Female	Male	Female				
1	Australia	1980	Risk Factor Prevalence Study	25-64	25-64	2,756	2,781	2	No	Standard	1
2	Australia	1983	Risk Factor Prevalence Study	25-64	25-64	3,733	3,812	2	No	Standard	1
3	Australia	1989	Risk Factor Prevalence Study	20-69	20-69	4,551	4,727	2	Unknown	Standard	1
4	Australia	1995-1996	National Nutrition Study	16+	16+	5,227	5,700	2 or 3	Yes	Standard	2
5	Australia	1999-2000	The Australian Diabetes, Obesity and Lifestyle Study (AusDiab)	25+	25+	5,023	6,113	3	Yes	Standard and Digital	
6	Australia	2004-2005	The Australian Diabetes, Obesity and Lifestyle Study (AusDiab)	30+	30+	2,885	3,478	3	Yes	Digital	
7	Australia	2011-2012	Australian Health Survey	10+	10+	11,290	12,309	2 or 3	Yes	Digital	2
8	Australia	2012	The Australian Diabetes, Obesity and Lifestyle Study (AusDiab)	37+	37+	2,052	2,536	3	Yes	Digital	
9	Canada	1981	Canada Fitness Survey	10-69	10-69	5,630	6,197	2	Yes	Standard	
10	Canada	1986-1992	Canada Heart Health Survey	18-74	18-74	11,353	11,737	2 to 4	Yes	Standard	
11	Canada	2007-2009	Canadian Health Measures Survey, Cycle 1	6-79	6-79	2,701	2,893	6 to 12	Yes	Digital	
12	Canada	2009-2011	Canadian Health Measures Survey, Cycle 2	6-79	6-79	2,764	3,019	6 to 12	Yes	Digital	
13	Canada	2012-2013	Canadian Health Measures Survey, Cycle 3	6-79	6-79	2,594	2,619	6 to 12	Yes	Digital	
14	Canada	2014-2015	Canadian Health Measures Survey, Cycle 4	6-79	6-79	2,603	2,602	6 to 12	Yes	Digital	
15	Canada	2016-2017	Canadian Health Measures Survey, Cycle 5	6-79	6-79	2,588	2,589	6 to 12	Yes	Digital	
16	Finland	1997	The National FINRISK Study	25-74	25-74	4,248	4,184	2	Yes	Standard	
17	Finland	2000-2001	Health 2000 Survey	30+	30+	2,870	3,463	2	Yes	Standard	
18	Finland	2002	The National FINRISK Study	25-74	25-74	3,267	3,814	3	Yes	Standard	
19	Finland	2007	The National FINRISK Study	25-74	25-74	2,926	3,310	3	Yes	Standard	
20	Finland	2008	Control group for Finnish male former elite athletes	61+		207		2	No	Standard	
21	Finland	2011	Young Finns Study	34-49	34-49	865	1,057	3	Yes	Random-zero	
22	Finland	2011-2012	Health 2011 Survey	30+	30+	2,061	2,563	2	Yes	Standard	
23	Finland	2012	The National FINRISK Study	25-74	25-74	2,771	3,042	3	Yes	Standard	
24	Finland	2017	The FinHealth Survey	18+	18+	2,677	3,115	3	Yes	Standard	
25	Germany	1997-1999	German National Health Interview and Examination Survey (GNHIES98)	18-79	18-79	3,445	3,616	3	Yes	Standard	
26	Germany	2008-2011	German Health Interview and Examination Survey for adults (DEGS1)	18-79	18-79	3,405	3,665	3	Yes	Digital	
27	Ireland	2006-2007	Survey of Lifestye, Attitudes and Nutritional in Ireland	45+	45+	526	679	3	Yes	Digital	
28	Ireland	2009-2011	The Irish Longitudinal Study on Ageing	50+	50+	2,696	3,173	2	Yes	Digital	
29	Italy	1992-1993	Italian Longitudinal Study on Aging	65-84	65-84	1,828	1,669	3	Yes	Standard	
30	Italy	1995-1996	Italian Longitudinal Study on Aging	68-90	68-90	1,133	1,003	3	Yes	Standard	
31	Italy	1998-2002	Osservatorio Epidemiologico Cardiovascolare	35-74	35-74	4,878	4,772	2	Yes	Standard	
32	Italy	2000-2001	Italian Longitudinal Study on Aging	73-93	73-93	683	671	3	Yes	Standard	
33	Italy	2004-2005	Italian Project on the Epidemiolofy of Alzheimer's Disease	65-84	65-84	1,578	1,440	2	No	Standard	
34	Italy	2008-2012	Osservatorio Epidemiologico Cardiovascolare/Health Examination Survey	35-80	35-80	4,370	4,337	3	Yes	Standard	
35	Japan	1980	National Cardiovascular Survey	30+	30+	6,323	7,916	1	Yes	Standard	
36	Japan	1986	National Nutrition Survey	20+	20+	5,471	7,050	1	Yes	Standard	2
37	Japan	1987	National Nutrition Survey	20+	20+	4,485	6,485	1	Yes	Standard	2
38	Japan	1988	National Nutrition Survey	20+	20+	4,528	6,254	1	Yes	Standard	2
39	Japan	1989	National Nutrition Survey	20+	20+	4,066	5,548	1	Yes	Standard	2
40	Japan	1990	National Nutrition Survey and National Cardiovascular Survey	20+	20+	4,448	5,988	1	Yes	Standard	
41	Japan	1991	National Nutrition Survey	20+	20+	4,237	5,706	1	Yes	Standard	2
42	Japan	1992	National Nutrition Survey	20+	20+	3,879	5,307	1	Yes	Standard	2
43	Japan	1993	National Nutrition Survey	20+	20+	3,492	4,921	1	Yes	Standard	2
44	Japan	1994	National Nutrition Survey	20+	20+	3,342	4,783	1	Yes	Standard	2
45	Japan	1995	National Nutrition Survey	20+	20+	3,269	4,654	1	Yes	Standard	2
46	Japan	1996	National Nutrition Survey	20+	20+	3,152	4,476	1	Yes	Standard	2
47	Japan	1997	National Nutrition Survey	20+	20+	3,069	4,386	1	Yes	Standard	2
48	Japan	1998	National Nutrition Survey	20+	20+	3,281	4,681	1	Yes	Standard	2
49	Japan	1999	National Nutrition Survey	20+	20+	2,550	3,814	1	Yes	Standard	2
50	Japan	2000	National Nutrition Survey and National Cardiovascular Survey	20+	20+	2,690	3,788	2	Yes	Standard	
51	Japan	2001	National Nutrition Survey	20+	20+	2,465	3,754	2	Yes	Standard	2
52	Japan	2002	National Nutrition Survey	20+	20+	2,394	3,473	2	Yes	Standard	2
53	Japan	2003	National Health and Nutrition Survey	20+	20+	2,350	3,491	2	Yes	Standard	2
54	Japan	2004	National Health and Nutrition Survey	20+	20+	1,743	2,590	2	Yes	Standard	2
55	Japan	2005	National Health and Nutrition Survey	20+	20+	1,723	2,496	2	Yes	Standard	2
56	Japan	2006	National Health and Nutrition Survey	20+	20+	1,925	2,787	2	Yes	Standard	2

	Country	Data years	Survey/Study name/Citation	Age range as in the NCD-RisC database		Sample size		Number of blood pressure measurements taken	Whether multiple cuff sizes were available for measurement	Type of device used to measure blood pressure	Note
				Male	Female	Male	Female				
57	Japan	2007	National Health and Nutrition Survey	20+	20+	1,810	2,634	2	Yes	Standard	2
58	Japan	2008	National Health and Nutrition Survey	20+	20+	1,981	2,822	2	Yes	Standard	2
59	Japan	2009	National Health and Nutrition Survey	20+	20+	1,887	2,726	2	Yes	Standard	2
60	Japan	2010	National Health and Nutrition Survey	20+	20+	1,758	2,470	2	Yes	Standard	
61	Japan	2011	National Health and Nutrition Survey	20+	20+	1,652	2,326	2	Yes	Standard	2
62	Japan	2012	National Health and Nutrition Survey	20+	20+	6,163	8,875	2	Yes	Standard	2
63	Japan	2013	National Health and Nutrition Survey	20+	20+	1,487	2,061	2	Yes	Standard	2
64	Japan	2014	National Health and Nutrition Survey	20+	20+	1,555	2,141	2	Yes	Standard	2
65	Japan	2015	National Health and Nutrition Survey	20+	20+	1,433	2,091	2	Yes	Standard	2
66	South Korea	1998	Korea National Health and Nutrition Examination Survey	10+	10+	4,514	5,193	2	Yes	Standard	
67	South Korea	2001	Korea National Health and Nutrition Examination Survey	10+	10+	3,269	3,998	2	Yes	Standard	
68	South Korea	2005	Korea National Health and Nutrition Examination Survey	10+	10+	2,863	3,589	3	Yes	Standard	
69	South Korea	2007	Korea National Health and Nutrition Examination Survey	10+	10+	1,547	1,998	3	Yes	Standard	
70	South Korea	2008	Korea National Health and Nutrition Examination Survey	10+	10+	3,480	4,478	3	Yes	Standard	
71	South Korea	2009	Korea National Health and Nutrition Examination Survey	10+	10+	3,915	4,839	3	Yes	Standard	
72	South Korea	2010	Korea National Health and Nutrition Examination Survey	10+	10+	3,264	4,021	3	Yes	Standard	
73	South Korea	2011	Korea National Health and Nutrition Examination Survey	10+	10+	3,104	3,941	3	Yes	Standard	
74	South Korea	2012	Korea National Health and Nutrition Examination Survey	10+	10+	2,924	3,826	3	Yes	Standard	
75	South Korea	2013	Korea National Health and Nutrition Examination Survey	10+	10+	2,934	3,686	3	Yes	Standard	
76	South Korea	2014	Korea National Health and Nutrition Examination Survey	10+	10+	2,727	3,551	3	Yes	Standard	
77	South Korea	2015	Korea National Health and Nutrition Examination Survey	10+	10+	2,816	3,432	3	Yes	Standard	
78	South Korea	2016	Korea National Health and Nutrition Examination Survey	10+	10+	3,004	3,790	3	Yes	Standard	
79	Spain	1990	Blood pressure in Spain: distribution, awareness, control, and benefits of a reduction in average pressure. Hypertension 1998; 32: 998-1002	35-65	35-65	810	1,211	3	Yes	Random-zero	
80	Spain	2000-2001	Influence of childhood socioeconomic circumstances, height, and obesity on pulse pressure and systolic and diastolic blood pressure in older people. J Hum Hypertens 2006; 20: 73-82	60+	60+	1,408	2,461	6	Yes	Standard and Digital	
81	Spain	2008-2010	Study on Nutrition and Cardiovascular Risk in Spain (ENRICA)	18+	18+	5,792	6,414	5	Yes	Digital	
82	Spain	2015	Study on Nutrition and Cardiovascular Risk in Spain (ENRICA)	65+	65+	743	800	4	Yes	Digital	
83	New Zealand	2008-2009	New Zealand Adult Nutrition Survey	15+	15+	1,949	2,396	3	Yes	Digital	
84	New Zealand	2012-2013	New Zealand Health Survey	15+	15+	4,952	6,692	3	Yes	Digital	2
85	New Zealand	2013-2014	New Zealand Health Survey	18+	18+	5,307	6,692	3	Yes	Digital	
86	New Zealand	2014-2015	New Zealand Health Survey	18+	18+	5,406	6,677	3	Yes	Digital	
87	New Zealand	2015-2016	New Zealand Health Survey	18+	18+	5,469	6,801	3	Yes	Digital	
88	United Kingdom	1991-1992	Health Survey for England	16+	16+	2,951	3,319	3	Yes	Digital	
89	United Kingdom	1993	Health Survey for England	16+	16+	6,745	7,489	3	Yes	Digital	
90	United Kingdom	1994	Health Survey for England	16+	16+	6,223	7,178	3	Yes	Digital	
91	United Kingdom	1998	Health Survey for England	10+	10+	6,767	7,828	3	Yes	Digital	
92	United Kingdom	1999-2001	British Women's Heart and Health Study		60-79		3,801	2	Yes	Digital	
93	United Kingdom	2001	Health Survey for England	10+	10+	6,156	7,191	3	Yes	Digital	2
94	United Kingdom	2000-2001	National Diet and Nutrition Survey	19-64	19-64	797	939	3	Yes	Digital	2
95	United Kingdom	2002	Health Survey for England	10+	10+	4,818	5,725	3	Yes	Digital	2
96	United Kingdom	2003	Health Survey for England	10+	10+	5,566	6,683	3	Yes	Digital	
97	United Kingdom	2004-2005	English Longitudinal Study of Ageing	52+	52+	3,404	4,159	3	Yes	Digital	
98	United Kingdom	2005	Health Survey for England	10+	10+	3,518	4,239	3	Yes	Digital	
99	United Kingdom	2006	Health Survey for England	10+	10+	5,123	6,088	3	Yes	Digital	
100	United Kingdom	2007	Health Survey for England	10+	10+	2,427	2,879	3	Yes	Digital	2
101	United Kingdom	2008	Health Survey for England	10+	10+	5,170	6,194	3	Yes	Digital	2
102	United Kingdom	2009	Health Survey for England	10+	10+	1,450	1,733	3	Yes	Digital	
103	United Kingdom	2006-2010	MRC National Survey of Health and Development	60-65	60-65	1,065	1,151	2	Yes	Digital	
104	United Kingdom	2010	Health Survey for England	10+	10+	2,611	3,283	3	Yes	Digital	
105	United Kingdom	2008-2012	National Diet and Nutrition Survey	10+	10+	1,037	1,241	3	Yes	Digital	2
106	United Kingdom	2011	Health Survey for England	10+	10+	2,648	3,313	3	Yes	Digital	
107	United Kingdom	2012	Health Survey for England	10+	10+	2,548	3,196	3	Yes	Digital	
108	United Kingdom	2013	Health Survey for England	10+	10+	2,932	3,632	3	Yes	Digital	
109	United Kingdom	2014	Health Survey for England	10+	10+	2,657	3,168	3	Yes	Digital	
110	United Kingdom	2013-2014	National Diet and Nutrition Survey	10+	10+	623	844	3	Yes	Digital	2

	Country	Data years	Survey/Study name/Citation	Age range as in the NCD-RisC database		Sample size		Number of blood pressure measurements taken	Whether multiple cuff sizes were available for measurement	Type of device used to measure blood pressure	Note
				Male	Female	Male	Female				
111	United Kingdom	2015	Health Survey for England	10+	10+	2,503	3,099	3	Yes	Digital	
112	United Kingdom	2016	Health Survey for England	10+	10+	2,311	2,880	3	Yes	Digital	
113	United States of America	1976-1980	US National Health and Nutrition Examination Survey (NHANES) II	10-74	10-74	7,265	7,767	3	No	Standard	
114	United States of America	1988-1994	US National Health and Nutrition Examination Survey (NHANES) III	17+	17+	8,966	9,843	3	Yes	Standard	
115	United States of America	1999-2000	US National Health and Nutrition Examination Survey (NHANES)	10+	10+	3,316	3,276	3	Yes	Standard	
116	United States of America	2001-2002	US National Health and Nutrition Examination Survey (NHANES)	10+	10+	3,587	3,489	3	Yes	Standard	
117	United States of America	2003-2004	US National Health and Nutrition Examination Survey (NHANES)	10+	10+	3,376	3,215	3	Yes	Standard	
118	United States of America	2005-2006	US National Health and Nutrition Examination Survey (NHANES)	10+	10+	3,382	3,230	3	Yes	Standard	
119	United States of America	2007-2008	US National Health and Nutrition Examination Survey (NHANES)	10+	10+	3,468	3,432	3	Yes	Standard	
120	United States of America	2009-2010	US National Health and Nutrition Examination Survey (NHANES)	10+	10+	3,677	3,687	3	Yes	Standard	
121	United States of America	2011-2012	US National Health and Nutrition Examination Survey (NHANES)	10+	10+	3,318	3,269	3	Yes	Standard	
122	United States of America	2013-2014	US National Health and Nutrition Examination Survey (NHANES)	10+	10+	3,447	3,624	3	Yes	Standard	
123	United States of America	2015-2016	US National Health and Nutrition Examination Survey (NHANES)	10+	10+	3,386	3,488	3	Yes	Standard	

1. The proportion of population living in urban areas in Australia from 1980 to 1989 was 85.5% according to the United Nations World Urbanization Propspects 2018 (https://population.un.org/wup/). Therefore we considered these studies as reasonably representative of the national population
2. Hypertension diagnosis information was not available in this survey.

Appendix Table 3. Age-specific prevalence of hypertension and rates of awareness, treatment, and control in women and men aged 40-79 years. Data are from the latest national survey in each country. Numbers in parentheses are the 95% confidence intervals.

**Country (years)**	**Age group** (years)	**Hypertension Prevalence**	**Awareness**	**Treatment**	**Control**
**Women**					
Australia (2012)	40-49	15% (11%-18%)	62% (49%-76%)	43% (31%-56%)	30% (17%-43%)
	50-59	25% (21%-29%)	74% (68%-81%)	62% (55%-69%)	44% (35%-53%)
	60-69	48% (44%-52%)	77% (72%-82%)	69% (63%-75%)	40% (34%-47%)
	70-79	67% (61%-73%)	80% (76%-84%)	73% (68%-79%)	36% (28%-43%)
Canada (2016-2017)	40-49	13% (5%-21%)	74% (53%-96%)	65% (42%-88%)	62% (40%-83%)
	50-59	38% (22%-53%)	64% (42%-86%)	50% (34%-67%)	-*
	60-69	46% (40%-51%)	73% (58%-88%)	72% (57%-86%)	59% (43%-76%)
	70-79	61% (49%-72%)	78% (67%-89%)	76% (65%-88%)	57% (42%-72%)
Finland (2017)	40-49	20% (17%-24%)	69% (60%-78%)	42% (32%-52%)	26% (17%-35%)
	50-59	43% (39%-47%)	76% (71%-81%)	51% (45%-57%)	29% (23%-34%)
	60-69	66% (62%-70%)	76% (72%-80%)	58% (53%-63%)	30% (26%-35%)
	70-79	82% (79%-86%)	81% (77%-85%)	71% (66%-76%)	29% (24%-34%)
Germany (2008-2011)	40-49	17% (14%-21%)	80% (70%-89%)	61% (50%-71%)	52% (42%-63%)
	50-59	34% (30%-39%)	82% (76%-88%)	73% (66%-80%)	56% (48%-64%)
	60-69	61% (57%-66%)	91% (87%-94%)	84% (80%-89%)	64% (58%-70%)
	70-79	75% (70%-79%)	91% (87%-94%)	88% (84%-92%)	57% (50%-63%)
Ireland (2009-2011) †	40-49	29% (16%-41%)	27% (4%-49%)	16% (0%-34%)	3% (0%-8%)
	50-59	39% (36%-42%)	58% (54%-62%)	51% (46%-55%)	31% (26%-35%)
	60-69	53% (50%-56%)	65% (61%-69%)	59% (55%-64%)	34% (30%-39%)
	70-79	71% (67%-75%)	75% (70%-79%)	72% (67%-77%)	33% (28%-39%)
Italy (2008-2012)	40-49	15% (13%-17%)	57% (50%-65%)	36% (29%-44%)	23% (17%-30%)
	50-59	39% (36%-42%)	70% (66%-75%)	59% (54%-64%)	31% (27%-36%)
	60-69	61% (58%-64%)	80% (77%-83%)	72% (69%-76%)	34% (31%-38%)
	70-79	79% (76%-82%)	83% (80%-86%)	78% (75%-81%)	29% (25%-32%)
Japan (2015) Δ	40-49	16% (12%-19%)	43% (28%-57%)	26% (14%-38%)	13% (5%-21%)
	50-59	33% (28%-38%)	54% (45%-63%)	39% (30%-49%)	18% (11%-26%)
	60-69	47% (43%-51%)	68% (63%-73%)	56% (49%-62%)	30% (24%-36%)
	70-79	69% (65%-74%)	77% (72%-82%)	71% (65%-76%)	37% (30%-44%)
New Zealand (2015-2016)	40-49	20% (17%-22%)	65% (57%-72%)	45% (37%-52%)	20% (14%-27%)
	50-59	38% (34%-42%)	73% (68%-78%)	56% (51%-62%)	34% (28%-40%)
	60-69	54% (50%-58%)	76% (71%-80%)	66% (61%-71%)	35% (31%-40%)
	70-79	67% (63%-71%)	83% (80%-87%)	75% (70%-80%)	44% (39%-49%)
South Korea (2016)	40-49	12% (9%-15%)	49% (36%-62%)	45% (32%-58%)	39% (27%-52%)
	50-59	31% (27%-35%)	72% (65%-79%)	70% (63%-77%)	48% (41%-56%)
	60-69	46% (41%-51%)	80% (74%-85%)	79% (73%-85%)	56% (49%-64%)
	70-79	72% (68%-77%)	86% (81%-92%)	85% (79%-90%)	59% (52%-66%)

Country (years)	Age group (years)	Hypertension Prevalence	Awareness	Treatment	Control
Spain (2015) ǂ	40-49	16% (14%-19%)	55% (47%-63%)	40% (32%-48%)	23% (17%-29%)
	50-59	33% (30%-37%)	66% (60%-72%)	51% (44%-58%)	33% (27%-40%)
	60-69	49% (41%-56%)	73% (63%-82%)	62% (52%-72%)	31% (21%-40%)
	70-79	63% (59%-67%)	74% (69%-79%)	63% (57%-68%)	26% (21%-31%)
UK (2016)	40-49	17% (14%-21%)	58% (44%-71%)	46% (33%-59%)	36% (23%-49%)
	50-59	28% (23%-32%)	63% (53%-73%)	53% (43%-64%)	35% (25%-46%)
	60-69	47% (42%-51%)	71% (64%-78%)	58% (51%-66%)	39% (30%-47%)
	70-79	64% (58%-69%)	80% (75%-86%)	70% (63%-77%)	38% (31%-45%)
USA (2015-2016)	40-49	20% (14%-26%)	78% (65%-91%)	67% (54%-81%)	49% (36%-61%)
	50-59	39% (34%-44%)	86% (79%-94%)	79% (71%-87%)	56% (48%-64%)
	60-69	59% (52%-67%)	89% (83%-95%)	83% (74%-93%)	61% (51%-72%)
	70-79	73% (67%-79%)	86% (81%-91%)	84% (79%-89%)	44% (38%-51%)
**Men**					
Australia (2012)	40-49	20% (15%-26%)	51% (40%-62%)	32% (19%-46%)	25% (12%-37%)
	50-59	36% (31%-42%)	67% (60%-73%)	51% (45%-57%)	29% (22%-36%)
	60-69	54% (49%-59%)	72% (67%-77%)	62% (56%-68%)	30% (23%-36%)
	70-79	66% (61%-71%)	73% (68%-79%)	68% (61%-74%)	30% (23%-37%)
Canada (2016-2017)	40-49	10% (4%-16%)	90% (74%-100%)	71% (52%-89%)	62% (43%-81%)
	50-59	28% (18%-37%)	74% (53%-94%)	72% (52%-93%)	63% (38%-87%)
	60-69	53% (47%-58%)	88% (74%-100%)	87% (74%-100%)	72% (59%-86%)
	70-79	62% (53%-70%)	86% (80%-91%)	85% (80%-91%)	71% (61%-81%)
Finland (2017)	40-49	37% (33%-42%)	69% (62%-76%)	28% (21%-35%)	14% (8%-19%)
	50-59	56% (52%-60%)	72% (67%-77%)	52% (46%-58%)	22% (17%-27%)
	60-69	70% (67%-74%)	76% (71%-80%)	61% (57%-66%)	31% (27%-36%)
	70-79	76% (71%-80%)	79% (74%-84%)	71% (66%-77%)	32% (27%-38%)
Germany (2008-2011)	40-49	26% (22%-31%)	67% (58%-77%)	45% (34%-55%)	24% (17%-32%)
	50-59	42% (38%-47%)	78% (73%-84%)	62% (55%-69%)	43% (37%-50%)
	60-69	59% (54%-64%)	88% (84%-92%)	80% (76%-85%)	60% (53%-67%)
	70-79	74% (69%-78%)	88% (84%-93%)	84% (78%-89%)	59% (52%-65%)
Ireland (2009-2011) †	40-49	51% (39%-63%)	25% (10%-40%)	17% (5%-29%)	1% (0%-3%)
	50-59	51% (48%-54%)	50% (45%-54%)	40% (36%-44%)	20% (17%-24%)
	60-69	64% (61%-68%)	57% (53%-61%)	52% (48%-56%)	27% (23%-30%)
	70-79	69% (65%-73%)	67% (62%-72%)	63% (58%-69%)	31% (26%-36%)
Italy (2008-2012)	40-49	35% (32%-38%)	51% (46%-56%)	27% (23%-32%)	9% (6%-12%)
	50-59	55% (52%-58%)	68% (64%-72%)	51% (47%-55%)	20% (17%-24%)
	60-69	70% (67%-72%)	76% (73%-79%)	68% (65%-71%)	32% (29%-35%)
	70-79	77% (74%-80%)	78% (75%-81%)	73% (70%-77%)	29% (26%-33%)
Japan (2015) [Δ]	40-49	35% (29%-42%)	41% (28%-54%)	29% (19%-40%)	10% (3%-18%)
	50-59	55% (47%-63%)	65% (57%-72%)	50% (40%-60%)	22% (14%-31%)
	60-69	68% (64%-72%)	71% (66%-76%)	52% (46%-58%)	23% (18%-29%)
	70-79	72% (67%-76%)	77% (72%-81%)	71% (66%-77%)	37% (31%-43%)

**Country (years)**	**Age group** (years)	**Hypertension Prevalence**	**Awareness**	**Treatment**	**Control**
New Zealand (2015-2016)	40-49	29% (25%-32%)	50% (42%-57%)	30% (23%-36%)	14% (9%-19%)
	50-59	38% (34%-42%)	69% (63%-75%)	50% (44%-56%)	26% (21%-32%)
	60-69	62% (58%-66%)	74% (69%-78%)	62% (57%-67%)	31% (26%-35%)
	70-79	66% (61%-71%)	77% (72%-82%)	71% (66%-77%)	38% (32%-44%)
South Korea (2016)	40-49	31% (26%-35%)	40% (31%-48%)	33% (25%-41%)	21% (14%-28%)
	50-59	42% (37%-47%)	66% (59%-73%)	62% (54%-69%)	40% (32%-48%)
	60-69	56% (50%-62%)	85% (80%-90%)	83% (78%-88%)	65% (59%-72%)
	70-79	65% (59%-71%)	90% (86%-94%)	87% (83%-92%)	62% (54%-69%)
Spain (2015) ǂ	40-49	33% (30%-36%)	46% (40%-52%)	27% (22%-32%)	14% (10%-17%)
	50-59	55% (51%-60%)	63% (58%-68%)	47% (42%-53%)	25% (20%-30%)
	60-69	72% (66%-77%)	72% (65%-79%)	62% (55%-69%)	30% (23%-36%)
	70-79	69% (65%-74%)	74% (69%-79%)	69% (63%-74%)	30% (25%-36%)
UK (2016)	40-49	20% (16%-25%)	58% (44%-73%)	36% (22%-51%)	27% (13%-41%)
	50-59	39% (34%-44%)	60% (51%-70%)	45% (36%-55%)	28% (19%-36%)
	60-69	53% (48%-58%)	66% (58%-73%)	59% (51%-66%)	38% (31%-45%)
	70-79	63% (57%-68%)	81% (75%-86%)	73% (67%-79%)	51% (44%-59%)
USA (2015-2016)	40-49	32% (25%-40%)	68% (59%-76%)	56% (48%-65%)	44% (36%-52%)
	50-59	44% (36%-52%)	80% (71%-88%)	63% (54%-73%)	48% (38%-57%)
	60-69	57% (51%-62%)	82% (73%-92%)	78% (69%-87%)	52% (43%-62%)
	70-79	55% (45%-65%)	88% (82%-94%)	84% (77%-91%)	53% (41%-65%)

* Hypertension control rate for women aged 50-59 years in Canada in 2016-2017 was unavailable due to Canadian regulations on releasing data on outcomes with small numbers.

† The latest national survey in Ireland had data for 50 to 79 years; data from an earlier survey in 2007 were used for 40 to 49 years.

ǂ The latest national survey in Spain had data for 60 to 79 years; data from an earlier survey in 2009 were used for 40 to 59 years.

[Δ] The question on awareness was not asked in 2015 in Japan; awareness data from 2010 were used.

Appendix Table 4. Change in hypertension prevalence since 2005 by country, sex and age group.

Country	Age group (years)	Women		Men	
		Average change * (percentage points per decade)	p-value for trend	Average change * (percentage points per decade)	p-value for trend
Australia	40-49	+10.7	0.4172	+8.1	0.4347
	50-59	+4.7	0.7824	+16.4	0.4466
	60-69	+11.2	0.5318	+10.5	0.4264
	70-79	+7.0	0.6690	+8.7	0.5779
Canada	40-49	+1.9	0.5506	-8.1	0.2683
	50-59	+15.5	0.0976	+1.5	0.8749
	60-69	-4.3	0.6128	-2.2	0.7999
	70-79	+2.7	0.6117	+2.5	0.7805
Finland	40-49	-7.0	0.3363	-7.8	0.5444
	50-59	-6.7	0.2249	-2.2	0.6832
	60-69	-8.1	0.2415	-5.0	0.5978
	70-79	+5.4	0.4828	+0.6	0.9427
Japan	40-49	-4.1	0.0615	-5.0	0.2245
	50-59	-7.9	0.0033	+5.3	0.2464
	60-69	-3.4	0.3346	+5.2	0.0197
	70-79	-2.6	0.2498	-2.9	0.3533
New Zealand	40-49	-0.1	0.9781	+0.8	0.8630
	50-59	+1.0	0.8243	-14.5	0.0344
	60-69	+1.8	0.7224	+3.2	0.0660
	70-79	-13.4	0.0595	+2.5	0.4636
South Korea	40-49	+0.1	0.9633	+5.7	0.0679
	50-59	-5.8	0.0147	-0.1	0.9587
	60-69	-2.4	0.5040	+3.7	0.4104
	70-79	+6.7	0.0224	+16.3	0.0002
United Kingdom	40-49	-3.1	0.2298	-2.7	0.2938
	50-59	-6.4	0.0143	-0.7	0.8368
	60-69	-3.6	0.2942	-5.5	0.0761
	70-79	-4.8	0.1857	-1.1	0.7471
United States of America	40-49	-0.7	0.7933	+4.4	0.3428
	50-59	-3.3	0.2196	+6.3	0.0783
	60-69	-3.5	0.2908	-3.6	0.2948
	70-79	+0.5	0.8871	-5.9	0.4611

* Average change is reported by fitting a linear regression for each age group and sex to the data in countries with more than three surveys since 2005.

Appendix Table 5. Changes in hypertension awareness, treatment, and control since 2005 by country, sex and age group.

Country	Age group (years)	Women		Men	
		Average change * (percentage points per decade)	p-value for trend	Average change * (percentage points per decade)	p-value for trend
**Awareness**					
Australia	40-49	-	-	-	-
	50-59	-	-	-	-
	60-69	-	-	-	-
	70-79	-	-	-	-
Canada	40-49	-	-	-0.9	0.9773
	50-59	-	-	-6.8	0.0478
	60-69	-13.9	0.1870	+2.8	0.6275
	70-79	-8.3	0.1868	-3.3	0.4136
Finland	40-49	-3.3	0.8631	+9.8	0.6899
	50-59	+4.0	0.7744	+4.6	0.8492
	60-69	+1.1	0.8884	+0.5	0.9542
	70-79	+4.8	0.6846	+10.9	0.1069
Japan	40-49	-	-	-	-
	50-59	-	-	-	-
	60-69	-	-	-	-
	70-79	-	-	-	-
New Zealand	40-49	+9.1	0.4290	-0.7	0.9490
	50-59	-1.8	0.8742	+22.0	0.1816
	60-69	+1.4	0.8580	+9.5	0.1900
	70-79	+8.4	0.2845	+5.8	0.3593
South Korea	40-49	-1.6	0.8323	-2.0	0.7668
	50-59	-6.7	0.2037	-2.8	0.6181
	60-69	-1.2	0.7098	+13.7	0.0154
	70-79	+11.7	0.0006	+20.4	0.0001
United Kingdom	40-49	-1.1	0.8608	+9.8	0.2572
	50-59	+10.1	0.0973	+7.7	0.2574
	60-69	+1.3	0.7887	+6.3	0.2578
	70-79	+9.3	0.0723	+14.4	0.0421
United States of America	40-49	-3.9	0.6437	-3.7	0.5945
	50-59	-1.3	0.6780	+9.5	0.1553
	60-69	+8.3	0.0561	+0.5	0.8788
	70-79	+4.6	0.2094	+0.4	0.8197

* Average change is reported by fitting a linear regression for each age group and sex to the data in countries with more than three surveys since 2005.

Appendix Table 5. (Continued)

Country	Age group (years)	Women		Men	
		Average change * (percentage points per decade)	p-value for trend	Average change * (percentage points per decade)	p-value for trend
**Treatment**					
Australia	40-49	-	-	-	-
	50-59	-	-	-	-
	60-69	-	-	-	-
	70-79	-	-	-	-
Canada	40-49	-	-	-0.7	0.9727
	50-59	-	-	-3.7	0.3544
	60-69	-16.7	0.0845	+3.1	0.5853
	70-79	-9.3	0.1964	-3.1	0.4189
Finland	40-49	+11.1	0.6558	+6.3	0.7560
	50-59	+6.7	0.3251	+12.3	0.2028
	60-69	+3.5	0.5108	+4.5	0.1072
	70-79	+11.4	0.0120	+15.9	0.0909
Japan	40-49	+7.5	0.0631	+9.0	0.0790
	50-59	+5.5	0.2184	+10.9	0.0166
	60-69	+2.2	0.5361	+4.6	0.1755
	70-79	+8.4	0.0236	+10.3	0.0104
New Zealand	40-49	-2.9	0.8020	+11.9	0.4025
	50-59	+3.4	0.6118	+9.8	0.0859
	60-69	-7.3	0.4713	+10.2	0.0425
	70-79	+3.5	0.3749	+4.0	0.5700
South Korea	40-49	+0.3	0.9727	+5.0	0.4800
	50-59	-0.2	0.9613	+2.0	0.7331
	60-69	+1.9	0.5883	+17.7	0.0049
	70-79	+14.6	0.0002	+22.0	0.0001
United Kingdom	40-49	+1.0	0.9264	+12.1	0.1453
	50-59	+9.9	0.0225	+8.8	0.0609
	60-69	+2.3	0.5399	+3.5	0.5167
	70-79	+10.6	0.0536	+16.0	0.0116
United States of America	40-49	+3.0	0.7523	-1.3	0.8859
	50-59	-1.0	0.8729	+4.7	0.4645
	60-69	+8.2	0.0257	-0.7	0.7926
	70-79	+5.4	0.1107	+0.9	0.7992

* Average change is reported by fitting a linear regression for each age group and sex to the data in countries with more than three surveys since 2005.

Appendix Table 5. (Continued)

Country	Age group (years)	Women		Men	
		Average change * (percentage points per decade)	p-value for trend	Average change * (percentage points per decade)	p-value for trend
**Control**					
Australia	40-49	-	-	-	-
	50-59	-	-	-	-
	60-69	-	-	-	-
	70-79	-	-	-	-
Canada	40-49	-22.9	0.3961	+2.7	0.9225
	50-59	-19.0	0.3969	-5.1	0.4778
	60-69	-11.3	0.2174	-2.0	0.7146
	70-79	+8.8	0.0958	+0.3	0.9646
Finland	40-49	+8.1	0.7438	+2.8	0.8939
	50-59	+10.0	0.3502	+9.0	0.0059
	60-69	+12.6	0.0966	+14.3	0.0222
	70-79	+14.2	0.0123	+19.9	0.1308
Japan	40-49	+8.2	0.0708	+6.6	0.0995
	50-59	+9.7	0.0173	+10.2	0.0046
	60-69	+10.4	0.0101	+10.6	0.0010
	70-79	+15.5	0.0003	+14.8	0.0007
New Zealand	40-49	+4.3	0.3728	+6.3	0.3998
	50-59	+4.3	0.0546	+5.6	0.2252
	60-69	-9.1	0.4045	-2.5	0.4526
	70-79	+10.8	0.1312	-1.8	0.8880
South Korea	40-49	+6.6	0.5161	+5.9	0.3074
	50-59	+3.6	0.3327	+2.2	0.7369
	60-69	+6.5	0.3497	+23.9	0.0016
	70-79	+17.7	0.0031	+31.2	0.0019
United Kingdom	40-49	+5.6	0.5257	+11.7	0.0689
	50-59	+10.3	0.0214	+12.6	0.0115
	60-69	+9.6	0.0015	+6.8	0.1039
	70-79	+13.2	0.0057	+18.8	0.0007
United States of America	40-49	+3.9	0.6743	+6.6	0.3342
	50-59	+7.8	0.4159	+4.4	0.4174
	60-69	+13.2	0.0612	+1.2	0.6719
	70-79	-2.8	0.7591	-1.5	0.7802

* Average change is reported by fitting a linear regression for each age group and sex to the data in countries with more than three surveys since 2005.

Appendix Table 6. Current and past clinical guidelines for hypertension in the 12 countries included in the analysis.

Guideline	Year	Threshold for immediate treatment	Initiating treatment based on CVD risk	Lifestyle modifications before initiating treatment†	Treatment target range (no other complications)‡	First line treatment	Note
			**Australia**				
NHFA	2004	BP ≥180/110	BP of 140-179/90-109 with associated clinical conditions, target organ disease, 5-year CVD risk ≥10%: (depending on risk) after lifestyle modifications<br><br>BP of 150-179/95-109 with 5-year CVD risk <10%: consider treatment after lifestyle modifications	BP of 140-179/90-109 with 5-year CVD risk <15%: for 3-12 months	BP <140/90 for ≥65 years<br>BP <130/85 for <65 years	Thiazide diuretic, beta-blocker, ACEI, CCB or ARB	Emphasised the role of lifestyle modifications as first-line intervention
NHFA	2008	BP ≥180/110, or<br>SBP ≥160 and DBP ≤70<br><br>BP ≥150/90, (depending on risk) after lifestyle modifications	regardless of BP with associated clinical conditions or end-organ damage<br><br>BP of 140-149 and DBP <90 with 5-year CVD risk ≥10%<br>after lifestyle modifications	BP of 140-179/90-109 with 5-year CVD risk ≤15%: for 3-12 months	BP <140/90	ACEI, ARB, CCB, or thiazide diuretic (≥65 years only)	Treatment recommendation is first stratified by CVD risk then by blood pressure
NHFA	2016	BP ≥160/100	regardless of BP with 5-year CVD risk >15%, or ≥10% with family history of premature CVD or aboriginal/Torres Strait Islander<br><br>BP of 140-159/90-99 with 5-year CVD risk ≥10%	BP of 130-139/85-89 with 5-year CVD risk 10-15%, no family history of premature CVD and not aboriginal/Torres Strait Islander: for 6 months then review BP<br><br>SBP of 140-159 with 5-year CVD risk <10%: for 2 months then review BP	BP <140/90	ACEI, ARB, CCB, thiazide diuretic<br><br>combination therapy for BP >20/10 over target	Treatment recommendation is first stratified by CVD risk then by blood pressure
			**Canada**				
CHS	1977	DBP ≥105	DBP of 90-104: based on clinical judgement	No recommendation	DBP <90	Not clearly recommended	None

Guideline	Year	Threshold for immediate treatment	Initiating treatment based on CVD risk	Lifestyle modifications before initiating treatment†	Treatment target range (no other complications)‡	First line treatment	Note
CHS	1984	DBP ≥100	DBP of 90-99 with target organ damage<br><br>DBP of 90-99 with no target organ damage, with other risk factors: based on clinical judgement	No recommendation	DBP <90	Thiazide diuretic, beta-blocker	None
CHS (elderly)	1986	BP ≥200/100 for 65-74 years<br><br>DBP ≥120 for ≥75 years	BP ≥180/90 with target organ damage (for 65-74 years)<br><br>BP ≥180/100 with target organ damage (for ≥75 years)	No recommendation	Reduce BP by 30/10-15	Thiazide diuretic	None
CHS	1993	DBP ≥100<br><br>SBP ≥160 and DBP <90 for ≥60 years	DBP of 90-99 with target organ damage<br><br>BP of 140-159/90-99 with no target organ damage: based on clinical judgement	No recommendation	DBP <90	Thiazide diuretic, beta-blocker	None
CHEP$^{\Delta}$	1999	BP ≥160/100 for <60 years<br><br>BP ≥160/105 for ≥60 years	BP ≥140/90 with target-organ damage, diabetes, renal disease, or CVD	No recommendation	BP <140/90	Thiazide diuretic, beta-blocker or ACEI	Combination therapy should be used if there is only a partial response to monotherapy
CHEP$^{\Delta}$	2008	BP ≥160/100	BP of 140-159/90-109 with macrovascular target organ damage (or other independent risk factors when DBP of 90-109)	No recommendation	BP <140/90	Thiazide diuretic, beta-blocker, ACEI, CCB, or ARB<br><br>Two agents if BP is 20/10 above target	None
CHEP$^{\Delta}$	2009	BP ≥160/100	BP of 140-159/90-109 with macrovascular target organ damage (or other independent risk factors when DBP of 90-109)	No recommendation	BP <140/90	Thiazide diuretic, beta-blocker (for <60 years), ACEI, CCB, or ARB<br><br>Two agents if BP is 20/10 above target	None

Guideline	Year	Threshold for immediate treatment	Initiating treatment based on CVD risk	Lifestyle modifications before initiating treatment†	Treatment target range (no other complications)‡	First line treatment	Note
CHEP[Δ]	2015	BP ≥160/100<br><br>SBP ≥160 for ≥80 years	BP of 140-159/90-109 with macrovascular target organ damage, diabetes, or other independent risk factors	No recommendation	BP <140/90<br><br>SBP <150 in very elderly (≥80 years)	Thiazide diuretic, beta-blocker (for <60 years), ACEI, CCB, or ARB<br><br>Two agents if BP is 20/10 above target	None
HC	2018	BP ≥160/100	BP of 140-159/90-109 with macrovascular target organ damage or other independent risk factors	No recommendation	BP <140/90	Monotherapy with thiazide or thiazide-like diuretic, beta-blocker (for <60 years), ARB, CCB or ACEI (for non-black)<br><br>Single-pill-combination of ACEI with CCB, ARB with CCB, or ACEI/ARB with diuretic	None
**Finland**							
FMSD/FHS	2002	BP ≥180/110<br><br>BP of 160-179/100-109, after lifestyle modifications	BP ≥140/90 with 10-year CVD risk ≥20%:<br>after lifestyle modifications	BP of 160-179/100-109: for 1 month	BP <140/85	Diuretic, beta-blocker, ACEI	None
FMSD/FHS	2005	BP ≥180/110<br><br>BP ≥160-179/100-109, lifestyle modifications	BP ≥140/90 with 10-year CVD risk ≥20%:<br>after lifestyle modifications	BP of 160-179/100-109: for 1 month	BP <140/85	ACEI, ARB, diuretic, CCB, beta-blocker	None
FMSD/FHS	2014	BP ≥180/110<br><br>BP of 140-179/90-109, after lifestyle modifications	No recommendation	BP of 140-179/90-109: for 1-2 months	BP <140/90	ACEI, ARB, diuretic, CCB	None
**Japan**							
MoH/JMA	1990	DBP ≥115<br>DBP of 90-114, after lifestyle modifications	No recommendation	DBP of 90-104 without organ damage: for 1-3 months<br><br>DBP of 105-114, SBP ≥160, or DBP ≥90 and SBP of 170-180 in the elderly	DBP <90, SBP of 140-160 for 60-69 years and 160-180 for ≥70 years	CCB, ACEI, diuretic, beta-blocker, alpha-blocker	None

Guideline	Year	Threshold for immediate treatment	Initiating treatment based on CVD risk	Lifestyle modifications before initiating treatment[†]	Treatment target range (no other complications)[‡]	First line treatment	Note
JSH	2000	BP ≥180/110<br><br>BP of 140-179/90-109, (depending on risk) after lifestyle modifications<br><br>BP ≥140-160/90 for 60-69<br>BP ≥160-170/90 for 70-79<br>BP ≥160-180/90 for 80-89 years	BP of 140-179/90-109 with diabetes, CVD or organ damage	BP of 140-179/90-109 without diabetes, CVD or organ damage: for 3-6 months	BP <130/85 in young and middle-aged<br><br>BP <140/90 for 60-69 years<br>BP <150-160/90 for 70-79 years<br>BP <160-170/90 for 80-89 years	CCB, ACEI, ARB, diuretic, beta-blocker, alpha-blocker	Risk stratification table available
JSH	2004	BP ≥180/110<br><br>BP of 140-179/90-109, (depending on risk) after lifestyle modifications	BP of 140-179/90-109 with diabetes, CKD, organ damage, CVD, or ≥3 risk factors	BP of 140-179/90-109 with low moderate risk: for 1-3 months	BP <130/85 in young/middle-aged<br><br>BP <140/90 in elderly	Diuretic, beta-blocker, CCB, ACEI, ARB, alpha-blocker, central sympathetic nerve inhibitor	None
JSH	2009	BP ≥180/110<br><br>BP of 140-179/90-109, (depending on risk) after lifestyle modifications	BP of 160-179/100-109 with 1-2 risk factors other than diabetes or MS meeting 3 items<br><br>BP of 140-179/90-109 with diabetes, CKD, organ damage, CVD, MS meeting 4 items, or ≥3 risk factors	BP of 140-179/90-109 with low moderate risk: for 1-3 months	BP <130/85 in young/middle-aged<br><br>BP <140/90 in elderly	Diuretic, beta-blocker, CCB, ACEI, ARB, alpha-blocker, central sympathetic nerve inhibitor	None
JSH	2014	BP ≥180/110<br><br>BP of 140-179/90-109, (depending on risk) after lifestyle modifications	BP of 160-179/100-109 with 1-2 risk factors other than diabetes or MS meeting 3 items<br><br>BP of 140-179/90-109 with diabetes, CKD, organ damage, CVD, MS meeting 4 items, or ≥3 risk factors	BP of 140-179/90-109 with low moderate risk: for 1-3 months	BP <140/90 for <75 years<br>BP <150/90 for ≥75 years	Diuretic, CCB, beta-blocker, ACEI or ARB	Lifestyle modifications are recommended as first-step treatment
**New Zealand**							
CHDSG	1993	BP ≥170/100	BP of 150-169/90-99 with 10-year CVD risk >20%: after lifestyle modifications	BP of 150-169/90-99 with 10-year CVD risk >20%: for 6 months	Lower by at least 10/5-6	Thiazide diuretic, beta-blocker	Cardiovascular guideline (first country to introduce a cardiovascular guideline)

Guideline	Year	Threshold for immediate treatment	Initiating treatment based on CVD risk	Lifestyle modifications before initiating treatment†	Treatment target range (no other complications)‡	First line treatment	Note
NZGG	2003	BP ≥170/100	5-year CVD risk >20%, or history of CVD<br><br>5-year CVD risk of 10-20% with no history of CVD: consider drug treatment after lifestyle modifications	5-year CVD risk of 10-20% with no history of CVD: for 3-6 months	BP <140/85	Thiazide diuretic, ACEI, beta-blocker, ARB or CCB	Cardiovascular guideline
NZ Primary Care Handbook 2012 (CVDRA update)	2013	BP ≥170/100	BP of 130-169/80-99 with 5-year CVD risk >20%, or known CVD<br><br>BP <130/80 with 5-year CVD risk >20%, or known CVD: initiate treatment cautiously<br><br>5-year CVD risk of 10-20%: discuss with patient about starting treatment	No recommendation	BP <140/85<br><br>No target for those with CVD risk <20%	Thiazide diuretic, beta-blocker, ACEI, ARB or CCB	Cardiovascular guideline
MoH	2018	BP ≥160/100, (depending on risk) after lifestyle modifications	BP ≥130/80 with 5-year CVD risk ≥15%<br><br>BP ≥140/90 with 5-year CVD risk of 5-15%: discuss with patient about starting treatment	BP ⩾160/100 with 5-year CVD risk <15%: no duration recommended	BP <130/80	ACEI, ARB, CCB, or thiazide diuretic	Cardiovascular guidance
				**South Korea**			
KSH	2000	BP ≥180/110<br><br>BP of 140-179/90-109, (depending on risk) after lifestyle modifications	No recommendation	Mild hypertension with low CVD risk: for 3-6 months	BP <140/90<br><br>BP <150/90 for elderly	Diuretics, beta-blocker, CCB, ACEI, ARB, alpha-blocker	Separate guideline for patients with CVD, diabetes or kidney disease
KSH	2004	BP ≥140/90, (depending on risk) after lifestyle modifications	More aggressive treatment for BP ≥140/90 with target organ damage or diabetes	BP of 140-149/90-99 with low CVD risk: for 3-6 months	BP <140/90	ACEI or beta-blocker for <55 years<br>CCB or diuretic for ≥55 years	Separate guideline for patients with CVD, diabetes or kidney disease

Guideline	Year	Threshold for immediate treatment	Initiating treatment based on CVD risk	Lifestyle modifications before initiating treatment†	Treatment target range (no other complications)‡	First line treatment	Note
KSH	2013	BP ≥160/100<br><br>BP ≥140-159/90-99, (depending on risk) after lifestyle modifications<br><br>SBP≥160 for ≥65 years	BP of 140-159/90-99 with 10-year CVD risk of 5-10%	BP of 130-139/85-89 with 10-year CVD risk <5%: for several weeks to 3 months	BP <140/90<br><br>SBP of 140-150 in the elderly	ACEI, beta-blocker (not for elderly), CCB, diuretic, ARB<br><br>Two drugs combination when BP ≥160/100 or 20/10 above target BP	Risk stratification table available<br><br>Separate guideline for patients with CVD, diabetes or kidney disease
KSH	2018	BP ≥160/100<br><br>BP of 130-159/85-99, (depending on risk) after lifestyle modifications<br><br>SBP≥160 for ≥65 years	BP of 140-159/90-99 with moderate to high risk	BP of 130-159/85-99 with 10-year CVD risk <5%: for several weeks to 3 months	BP <140/90 for low moderate risk or elderly<br><br>BP <130/80 for high risk	ACEI, ARB, beta-blocker, CCB, diuretics<br><br>Two drugs combination when BP ≥160/100 or 20/10 above target BP	Risk stratification table available<br><br>Separate guideline for patients with CVD, diabetes or kidney disease
**UK**							
BHS 1	1989	DBP ≥100	No recommendation	No recommendation	DBP ~85-90	Diuretic or beta-blocker	Observe patients with DBP of 95-99 every 3-6 months
BHS 2	1993	DBP ≥100 or BP ≥160/95	DBP of 90-99 with higher BP within the range, older age (>60 years), target organ damage or coexisting risk factors	No recommendation	BP <160/90	Thiazide diuretic or beta-blocker	Recommended new alternative drugs: calcium entry blockers, converting enzyme inhibitors and alpha-blockers
BHS 3	1999	BP ≥160/100	BP of 140-159/90-99 with target organ damage, CVD, diabetes or 10-year CHD risk ≥15%	BP of 140-159/90-99 with no target organ damage or CVD: for 4-6 months	BP <140/85<br>BP <150/90 minimal	Low dose thiazide diuretic or beta-blocker	None
BHS 4	2004	BP ≥160/100	BP of 140-159/90-99 with target organ damage, CVD, diabetes or 10-year CVD risk ≥20%	BP of 140-159/90-99 with no target organ damage or CVD: for up to 6 months	BP <140/85<br>BP <150/90 minimal	Younger (<55 years) and non-black: ACEI, ARB or beta-blocker<br><br>Older (≥55 years) or black: CCB or thiazide diuretic	Fixed dose combinations are recommended
NICE CG18	2004	BP ≥160/100	BP of 140-159/90-99 with target organ damage, CVD, 10-year CHD risk ≥15% or 10-year CVD risk ≥20%	No recommendation	BP <140/90	Low dose thiazide-like diuretics	Separate guideline for patients with diabetes

Guideline	Year	Threshold for immediate treatment	Initiating treatment based on CVD risk	Lifestyle modifications before initiating treatment†	Treatment target range (no other complications)‡	First line treatment	Note
NICE CG127	2011	BP ≥160/100	BP of 140-159/90-99 in younger ages (<80 years) with target organ damage, CVD, diabetes, renal disease or 10-year CVD risk ≥20% or equivalent	No recommendation	BP <140/90 for <80 years<br>BP <150/90 for ≥80 years	Younger (<55 years) and non-black: ACEI or ARB<br><br>Older (≥55 years) or black: CCB or thiazide-like diuretic if CCB not suitable	Treatment not recommended for BP of 140-159/90-99 for >80 years
NICE	under public consultation	BP ≥160/100	BP of 140-159/90-99 for <80 years with target organ damage, CVD, diabetes, renal disease or 10-year CVD risk ≥10%	No recommendation	BP <140/90 for <80 years<br>BP <150/90 for ≥80 years	Younger (<55 years) and non-black, or with diabetes: ACEI or ARB<br><br>Older (≥55 years) or black, and no diabetes: CCB or thiazide-like diuretic if CCB not suitable	Recommended to consider starting drug treatment for BP of 140-159/90-99 for >80 years
**USA**							
JNC 1	1976	DBP ≥105	DBP of 90-104 with consideration of other risk factors	No recommendation	No recommendation	Thiazide-type diuretic	SBP not included because the recommendations "would be far too complex"
JNC 2	1980	DBP ≥105	DBP of 90-104 with consideration of target organ damage, smoking, family history of premature CVD, elevated SBP, diabetes and elevated cholesterol	No recommendation	DBP <90<br>A limited goal acceptable in those with initial DBP≥105 if side effects are intolerable	Thiazide-type diuretic	None
JNC 3	1984	DBP ≥95	DBP of 90-94 with consideration of other risk factors	DBP of 90-94 or sustained SBP≥160, and low risk: carefully monitored but physicians can decide whether to treat	DBP <90<br>A limited goal acceptable in those with initial DBP≥105 if side effects are intolerable	Thiazide-type diuretic or beta-blocker	None
JNC 4	1988	DBP ≥95	DBP of 90-94 with high risk	DBP of 90-94 with low CVD risk or sustained SBP ≥160: carefully monitored but physicians can decide whether to treat	BP <140/90	Thiazide-type diuretic, beta-blocker, ACEI or calcium antagonist	None

Guideline	Year	Threshold for immediate treatment	Initiating treatment based on CVD risk	Lifestyle modifications before initiating treatment[†]	Treatment target range (no other complications)[‡]	First line treatment	Note
JNC 5	1993	BP ≥160/110<br><br>BP of 140-159/90-109, (depending on risk) after lifestyle modifications	BP of 140-149/90-94 with no other risks: physicians may choose not to treat	BP of 140-179/90-109 or sustained SBP ≥160 with no other risks: for 3-6 months	BP <140/90<br><br>For ISH:<br>SBP <160 when SBP≥180<br>Lowered by 20 mmHg when SBP of 160-179	Thiazide-type diuretic or beta-blocker	None
JNC 6	1997	BP ≥160/100<br><br>BP of 140-159/90-99, (depending on risk) after lifestyle modifications	BP of 130-139/85-89 with target organ damage, CVD or diabetes	BP of 140-159/90-99 without target organ damage or CVD or diabetes: for 6-12 months	BP <140/90	Diuretic or beta-blocker	Published risk stratification table for the first time
JNC 7	2003	BP ≥140/90 after lifestyle modifications	No recommendation	BP ≥140/90: no specified duration	BP <140/90	Thiazide-type diuretic (alone for most, or in combination with another class of drug) for BP in 140-159/90-99<br><br>Two-drug combination (thiazide-type diuretic with one of ACEI, ARB, beta-blocker or CCB) for BP ≥160/100	Recommended using fixed-dose combination when more than one drug is used
ASH/ISH	2013	BP ≥160/100<br><br>BP of 140-159/90-99, (depending on risk) after lifestyle modifications	BP ≥140/90 for ≥80 years with diabetes or kidney disease	BP of 140-159/90-99 with no other risks: delaying drug therapy but no specified duration	BP <140/90	Two drugs (CCB or thiazide diuretic + ACEI or ARB) for BP ≥160/100<br><br>CCB or thiazide diuretic for ≥60 years or black<br><br>ACEI or ARB for <60 years and non-black	None
JNC 8	2014	BP ≥140/90 for <60 years<br>BP ≥150/90 for ≥60 years	BP ≥140/90 with CKD or diabetes regardless of age	No recommendation	BP <140/90 for <60 years<br>BP <150/90 for ≥60 years	Thiazide-type diuretic, CCB, ACEI, or ARB for non-black<br>Thiazide-type diuretic or CCB for black	Removed recommendations on classifications of hypertension
ACC/AHA	2017	BP ≥140/90	BP in 130-139/80-89 with clinical ASCVD or 10-year CVD risk ≥10%	No recommendation	BP <130/80	Thiazide diuretic, CCB, ACEI or ARB<br><br>2 drugs of different classes for BP≥140/90 or BP of 20/10 over target	None

Guideline	Year	Threshold for immediate treatment	Initiating treatment based on CVD risk	Lifestyle modifications before initiating treatment[†]	Treatment target range (no other complications)[‡]	First line treatment	Note
**WHO** (used in many parts of Europe until 2003)							
WHO/ISH	1986	DBP ≥105<br><br>DBP of 95-104, after lifestyle modifications	DBP of 90-94 with high risk: consider treatment	DBP of 95-104: for 3-6 months	DBP <90	Diuretic or beta-blocker	None
WHO/ISH	1993	BP ≥180/105 or sustained BP ≥160/95	BP of 140-159/90-94 with other risk factors: consider treatment	BP of 140-179/90-104: for 1-7 months, depending on follow-up BP measurements every 3 months	BP in 120-130/80 in young patients with mild hypertension<br><br>BP <140/90 in others	Diuretic, beta-blocker, ACEI, CCB, alpha-blocker	None
WHO/ISH	1999	High and very high risk (10-year stroke or MI risk ≥20%) with BP ≥180/110, existing CVD, diabetes, target organ damage or ≥3 risk factors	BP ≥140/90 and medium risk (10-year risk of 15-20%) or BP ≥150/95 and low risk (10-year risk <15%),<br>after lifestyle modifications	Medium risk for 3-6 months<br><br>Low risk for 6-12 months	BP <130/85 in younger<br><br>BP <140/90 in older	No recommendation	Patients with BP of 140-149/90-94 may choose not to start drug treatment
WHO/ISH	2003	10-year stroke or MI risk ≥20% with BP ≥180/110, existing CVD, diabetes, target organ damage or ≥3 risk factors	Not clearly stated: BP ≥160/100 for both medium and low risk are recommended, but alluded the emerging evidence of the benefits for those with BP going down to 140/90	Not clearly stated	BP <130/80 in high risk<br>BP <150/90 in low risk and medium risk and <55 years<br>BP <140/90 in low risk and medium risk and ≥55 years	Low dose diuretic, unless compelling indication for another class of drugs	Last guideline before WHO stopped publishing hypertension guidelines in favour of risk-based treatment
**ESH/ESC** (adopted in Italy, Germany, Spain and parts of Ireland[§])							
ESH/ESC	2003	BP ≥180/110<br><br>BP of 140-179/90-109, (depending on risk) after lifestyle modifications	BP of 130-139/85-89 with ≥3 risk factors, target organ damage, diabetes or associated clinical conditions	BP of 140-179/90-109 with 0-2 risk factors: 3-12 months; treatment is optional for low risk	BP <140/90	Diuretic, beta-blocker, CCB, ACEI, or ARB	Risk stratification table available
ESH/ESC	2007	BP ≥180/110<br><br>BP of 140-179/90-109, (depending on risk) after lifestyle modifications	BP of 130-139/85-89 with CVD, renal disease, diabetes, ≥3 risk factors, organ damage or MS: (depending on risk) after lifestyle modifications<br><br>BP of 120-129/80-84 with CVD or renal disease	BP of 140-179/90-109: for several weeks to several months<br><br>BP of 130-139/85-89 with ≥3 risk factors, target organ damage or MS	BP <140/90<br><br>BP <130/80 in high risk	Thiazide diuretic, beta-blocker, ACEI, CCB, or ARB	Risk stratification table available

Guideline	Year	Threshold for immediate treatment	Initiating treatment based on CVD risk	Lifestyle modifications before initiating treatment†	Treatment target range (no other complications)‡	First line treatment	Note
ESH/ESC	2013	BP ≥180/110<br><br>BP of 140-179/90-109, (depending on risk) after lifestyle modifications	BP of 140-159/90-99 with organ damage, CKD stage ≥3, diabetes or CVD<br><br>BP of 160-179/100-109 with ≥3 risk factors, or organ damage, CKD stage ≥3, diabetes or CVD	BP of 140-159/90-99 without CVD, organ damage, CKD or diabetes: for several weeks to several months<br><br>BP of 160-179/90-99 with 0-2 risk factors: for several weeks	BP <140/90	Thiazide diuretic, beta-blocker, ACEI, CCB, or ARB	Risk stratification table available
ESC/ESH	2018	BP ≥180/110<br><br>BP of 140-179/90-109, (depending on risk) after lifestyle modifications	BP of 130-139/85-89 with very high risk due to CVD especially CAD	BP of 140-159/90-99 without CVD, hypertension-mediated organ damage or renal disease: for 3-6 months	DBP <80 for all<br><br>SBP of 120-129 for <65 years<br>SBP of 130-139 for ≥65 years	Two-drug combination except in frail old patients and those with BP of 140-159/90-99 and low risk	Risk stratification table available<br><br>Single-pill combination is preferable

Units for blood pressure are mmHg.
† Lifestyle modifications *alongside* pharmacological treatment are always recommended and they are not specified individually for each guideline in this table.
‡ Treatment targets usually differ for patients with other conditions, e.g., diabetes. These separate targets are not listed.
∆ CHEP publishes annual updates of hypertension guidelines. Only selected years with noticeable changes are listed in this table.
§ Both UK and ESH/ESC guidelines were used in Ireland.

BP: blood pressure; SBP: systolic blood pressure; DBP: diastolic blood pressure.
ASCVD: atherosclerotic cardiovascular disease; CAD: coronary artery disease; CKD: chronic kidney disease; CVD: cardiovascular disease; MI: myocardial infarction; MS: metabolic syndrome.
ACEI: angiotensin-converting-enzyme inhibitor; ARB: angiotensin receptor blocker; CCB: calcium channel blocker.
ACC: American College of Cardiology; AHA: American Heart Association; ASH: American Society of Hypertension; BHS: British Society of Hypertension; CHEP: Canadian Hypertension Education Program; CHDSG: Core Health & Disability Services Guidelines; CHS: Canadian Hypertension Society; CVDRA: Cardiovascular Disease Risk Assessment; ESC: European Society of Cardiology; ESH: European Society of Hypertension; FHS: Finnish Hypertension Society; FMSD: Finnish Medical Society Duodecim; HC: Hypertension Canada; ISH: International Society of Hypertension; JMA: Japan Medical Association; JNC: Joint National Committee; JSH: Japanese Society of Hypertension; KSH: Korean Society of Hypertension; MoH: Ministry of Health; NHFA: National Heart Foundation of Australia; NICE CG: The National Institute for Health and Care Excellence Clinical Guideline; NZGG: New Zealand Guidelines Group; WHO: World Health Organization.

Appendix Table 7. Selected characteristics of healthcare and insurance system in the 12 countries included in the analysis.

Country	Public and private insurance coverage	Financing of healthcare	Major change in healthcare and insurance system	Co-payment	Hypertension management in primary care	National screening and hypertension education programmes	Financial incentives to primary care physicians
Australia	Public 100%; optional private health cover	General taxation and Medicare Levy (2% of taxable income – some exemptions for low income earners)	Medicare (universal coverage) introduced in 1975 and refined in 1984	Co-payment and price cap on medicines; safety net on annual medicine spending	Hypertension is diagnosed and managed in primary care	None	Some incentives for managing chronic diseases
Canada	Public 100%, for physician consultations and for drugs provided in hospitals. Private benefit plans vary in coverage of prescriptions	Personal and corporate taxation at the federal and provincial level.	Canada Health Act 1984	Needed for prescriptions; private benefit plans cover varying proportions of prescriptions	Hypertension is diagnosed and managed in primary care	Canadian Hypertension Education Program 1999; Canadian Hypertension Awareness Program 2003; Hypertension Canada 2018	Provincial programmes exist but no national programme
Finland	Public 100%	General taxation	Public Health Law 1972 (every municipality became responsible for arranging primary care for residents)	Small co-payments and out-of-pocket payments. All healthcare costs capped at 683 euros per year	Hypertension is mainly diagnosed and managed in primary care	None	None
Germany	99.9% coverage (87.6% public, 11.5% private)	78% compulsory health insurance (equal contributions by employer and employee), 7% government schemes, 13% out-of-pocket, 1% voluntary health insurance	Federal Joint Committee 2004	Co-payment for outpatient prescriptions, for inpatient care in hospitals and rehabilitation centres, and for prescribed medical devices	Hypertension is diagnosed and managed in primary and specialist care	Health check-ups including BP screening for those aged ≥35 years since 1989 (expanding to ≥18 years in 2019)	None
Ireland	Health insurance does not cover care at GP level; 46% population had private health insurance (2015)	69% tax, 15% out-of-pocket payments, 13% private (2014)	Free GP care for those aged <6 years and >70 years (2011)	Out-of-pocket fees for access primary care, price set by self-employed GPs; exemption according to age and income	Hypertension is mainly diagnosed and managed in primary care	None	None
Italy	Public 100% National Health Service (SSN) since 1978; voluntary additional private cover	General taxation and contributions (central and regional governments)	National Health Service (SSN) in 1978	Partial payment per prescription; exemption for medicine and specialised tests in hypertensive patients if performed in specialist centres	Hypertension is diagnosed and managed in primary care and specialist centres	National Preventive Plan 2005-2008 recommended CVD risk assessment including BP measurements	None

Country	Public and private insurance coverage	Financing of healthcare	Major change in healthcare and insurance system	Co-payment	Hypertension management in primary care	National screening and hypertension education programmes	Financial incentives to primary care physicians
Japan	Social insurance 100%; voluntary private health insurance, participated by ~90% of households	Taxes (39% in 2014), insurance premiums (49%), out-of-pocket payments (12%)	Long-term care insurance system (2000); Integrated Community Care System (2006); late-stage medical care system for the elderly aged ≥75 years (2008); Comprehensive Reform of Society Security and Tax (2010); Regional Healthcare Vision (2014)	10-30% of costs	Hypertension is diagnosed and managed in primary care	Specific health check-up and specific health guidance for those aged 40-74 since 2008	A referral system for the use of hospital services through clinic services
New Zealand	Public 100%; 35% adults also had private insurance in 2017/18	General taxation (78% in 1999), supplemented by out-of-pocket payments (16%) and private health insurance (6%)	Introduced market model practices into health sector in the 1990s; returned responsibility to District Health Boards in Public Health and Disability Act 2000	Patients have co-payments for primary care consultations, varies by socioeconomic status, age, and ethnicity (e.g., free checks for Maori)	Cardiovascular risk assessment (including hypertension management) managed in primary care	None	Incentives for diabetes and cardiovascular risk assessments
South Korea	Public; increased from ~25% in 1980 to ~100% in 1989	56.5% public sources, 6.7% private insurance, 36.8% out of pocket in 2014	National Health Insurance established in 1989	Payment per prescription	Hypertension is diagnosed and managed in primary care	National health screening programme (including BP check) has been expanded especially for those aged ≥40 years since 2007; national comprehensive management plan (2006) for cardio- and cerebro-vascular diseases	None
Spain	Public (National Health Service); ~15% of the population also have private insurance	General taxation	Royal Decree Law 16/2012, 7/2018	Small co-payment to pharmacy	Hypertension is diagnosed and managed in primary care	Preventive Activities and Health Promotion Program (PAPPS) since 1989 recommended BP measurements in primary care every two years for those aged >40 years	None

Country	Public and private insurance coverage	Financing of healthcare	Major change in healthcare and insurance system	Co-payment	Hypertension management in primary care	National screening and hypertension education programmes	Financial incentives to primary care physicians
UK	Public (National Health Service) 100%; voluntary additional private cover	General taxation	None	Mostly free at point of use; co-payment for prescriptions with exemption for special groups	Hypertension is diagnosed and managed in primary care	NHS Health Check for those aged ≥40 years since 2009	Quality and Outcomes Framework (QOF): voluntary payment scheme that linked up to 25% of GP's income to performance
USA	Public and private insurance for ~80-90% of population	48% public source, 40% private insurance, 12% out of pocket	Affordable Care Act 2010	Dependent on health system and service	Hypertension is diagnosed and managed in primary care; testing and screening frequently done non-systematically outside the healthcare system	National High Blood Pressure Education Program since 1972	Dependent on health insurance scheme

BP: blood pressure; GP: general practitioner.

Appendix Figure 1. Trends in hypertension prevalence, and rates of awareness, treatment, control, and control among those treated, by country, sex and age group.

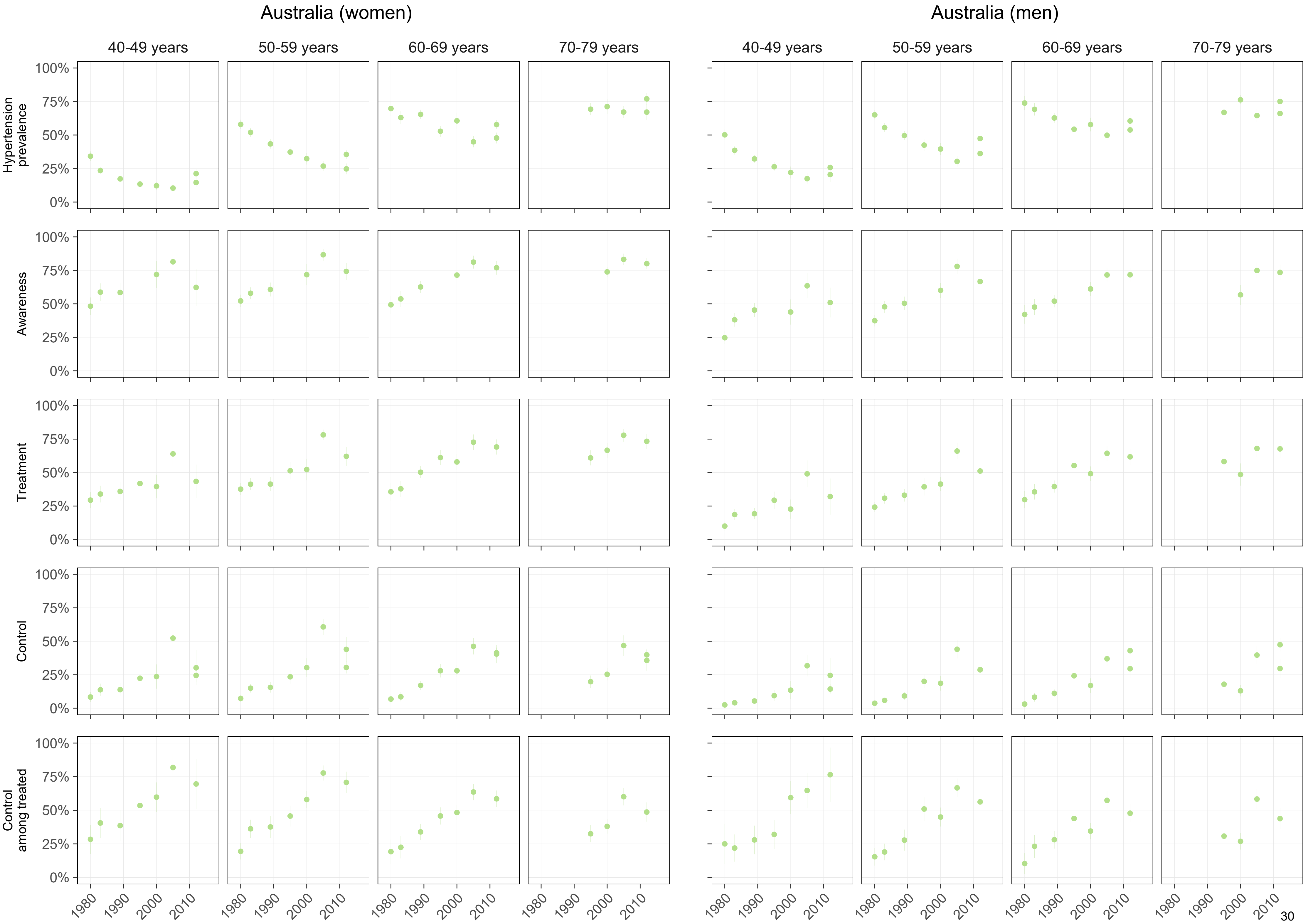

Australia (women)
Australia (men)
40-49 years
50-59 years
60-69 years
70-79 years
Hypertension prevalence
Awareness
Treatment
Control
Control among treated
0%
25%
50%
75%
100%
1980
1990
2000
2010

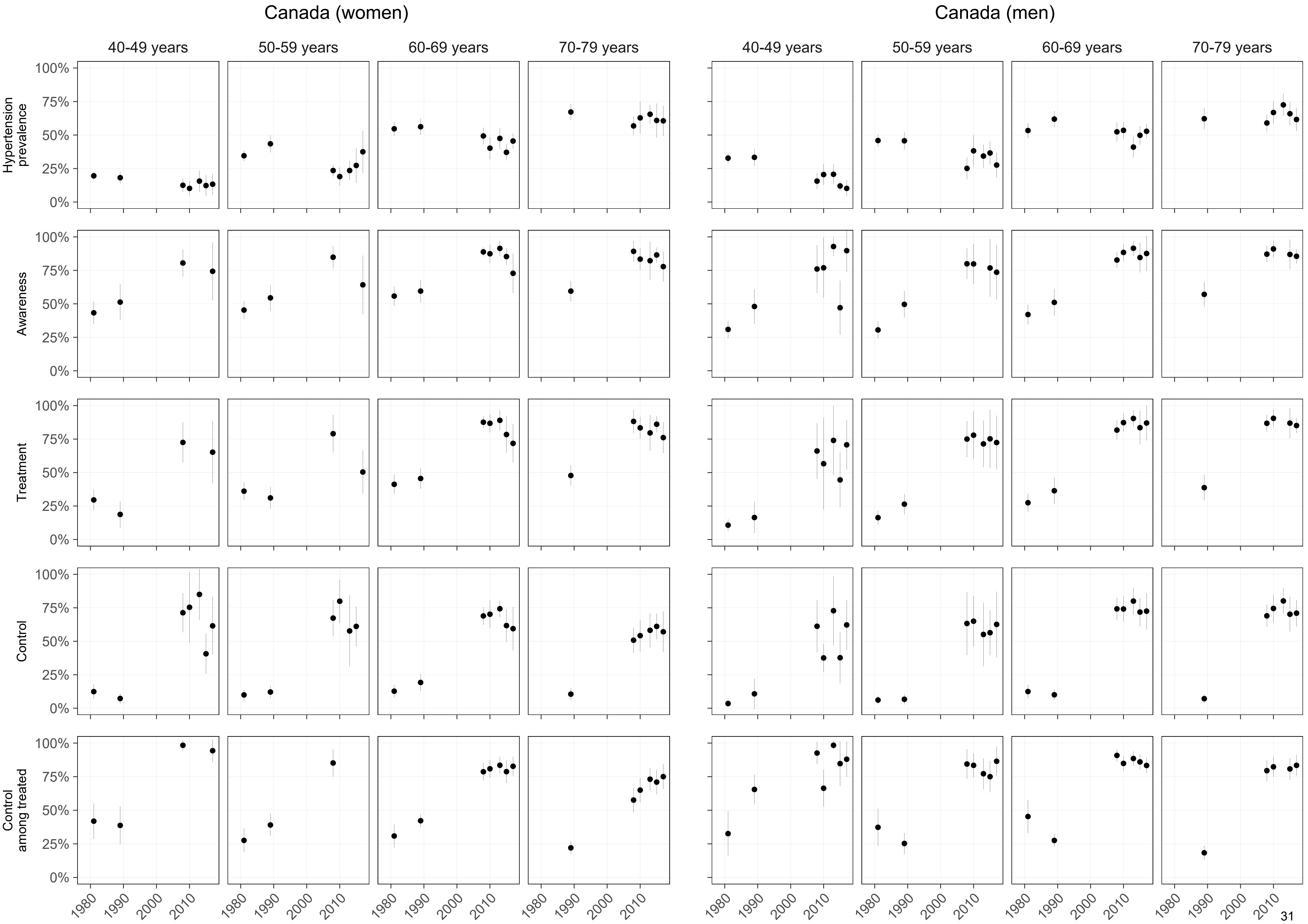

Canada (women)
Canada (men)
40-49 years
50-59 years
60-69 years
70-79 years
Hypertension prevalence
Awareness
Treatment
Control
Control among treated
0%
25%
50%
75%
100%
1980
1990
2000
2010

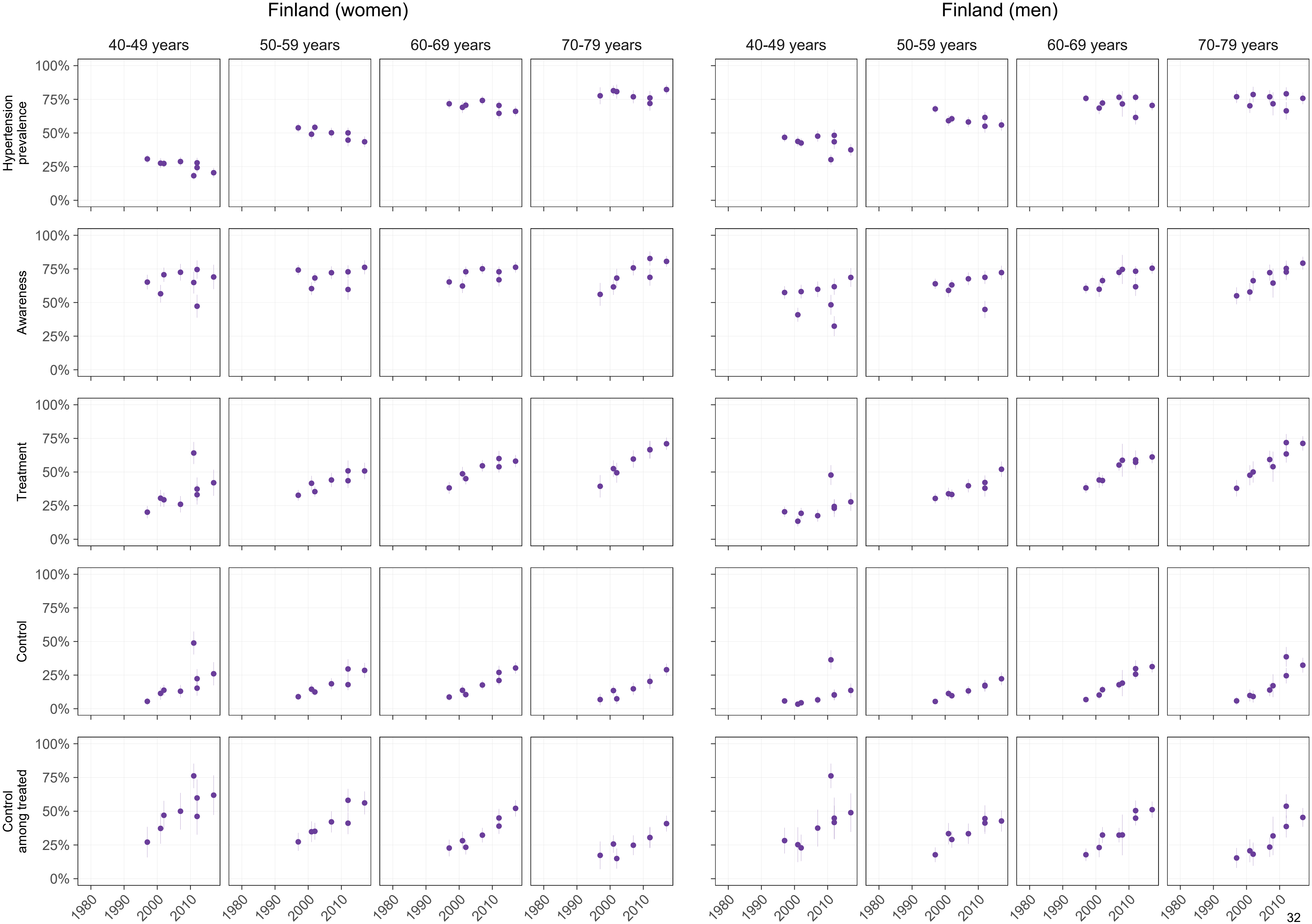

Finland (women)
Finland (men)
40-49 years
50-59 years
60-69 years
70-79 years
Hypertension prevalence
Awareness
Treatment
Control
Control among treated
100%
75%
50%
25%
0%
1980
1990
2000
2010

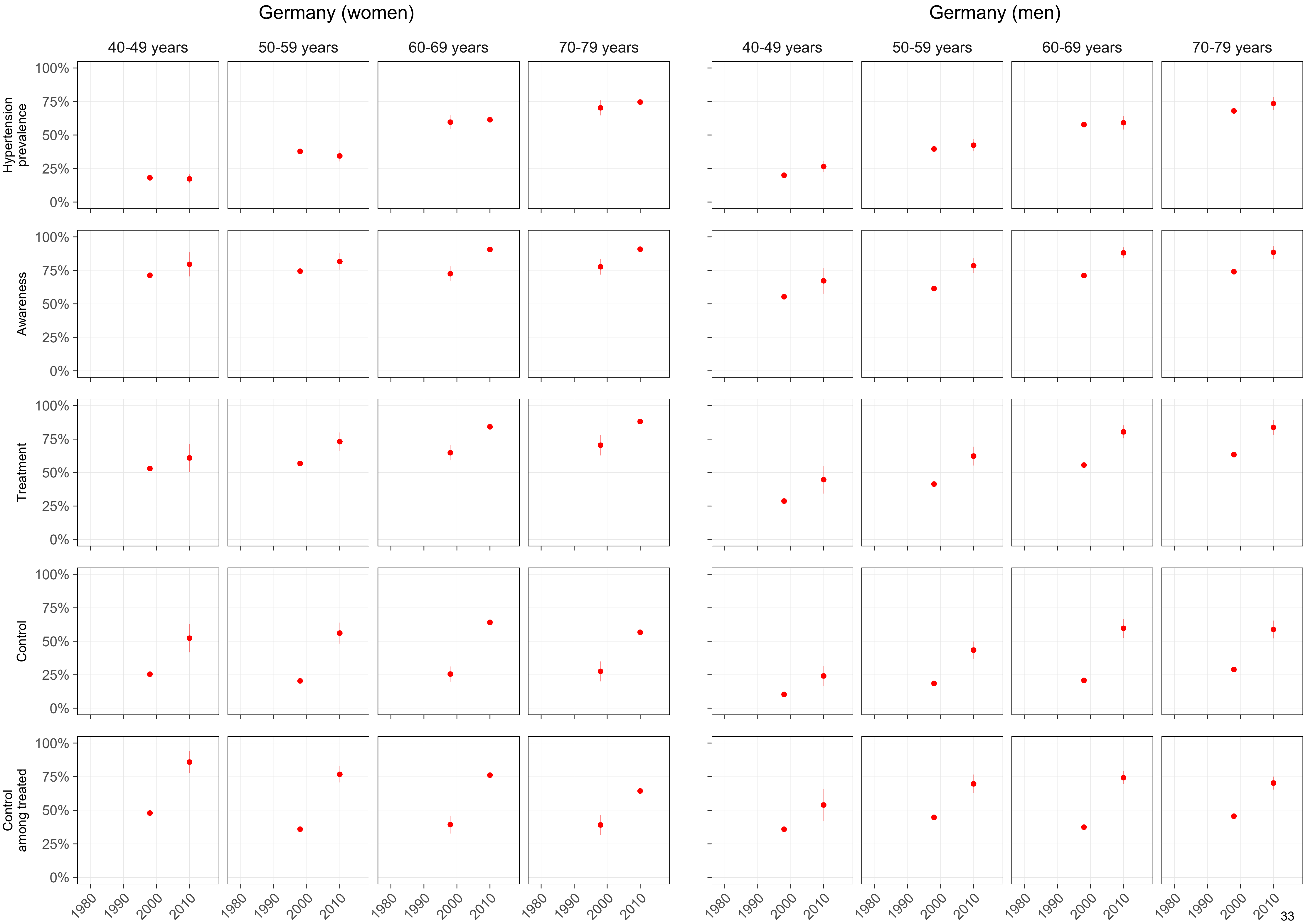
Germany (women)
Germany (men)
40-49 years
50-59 years
60-69 years
70-79 years
Hypertension prevalence
Awareness
Treatment
Control
Control among treated
0%
25%
50%
75%
100%
1980
1990
2000
2010

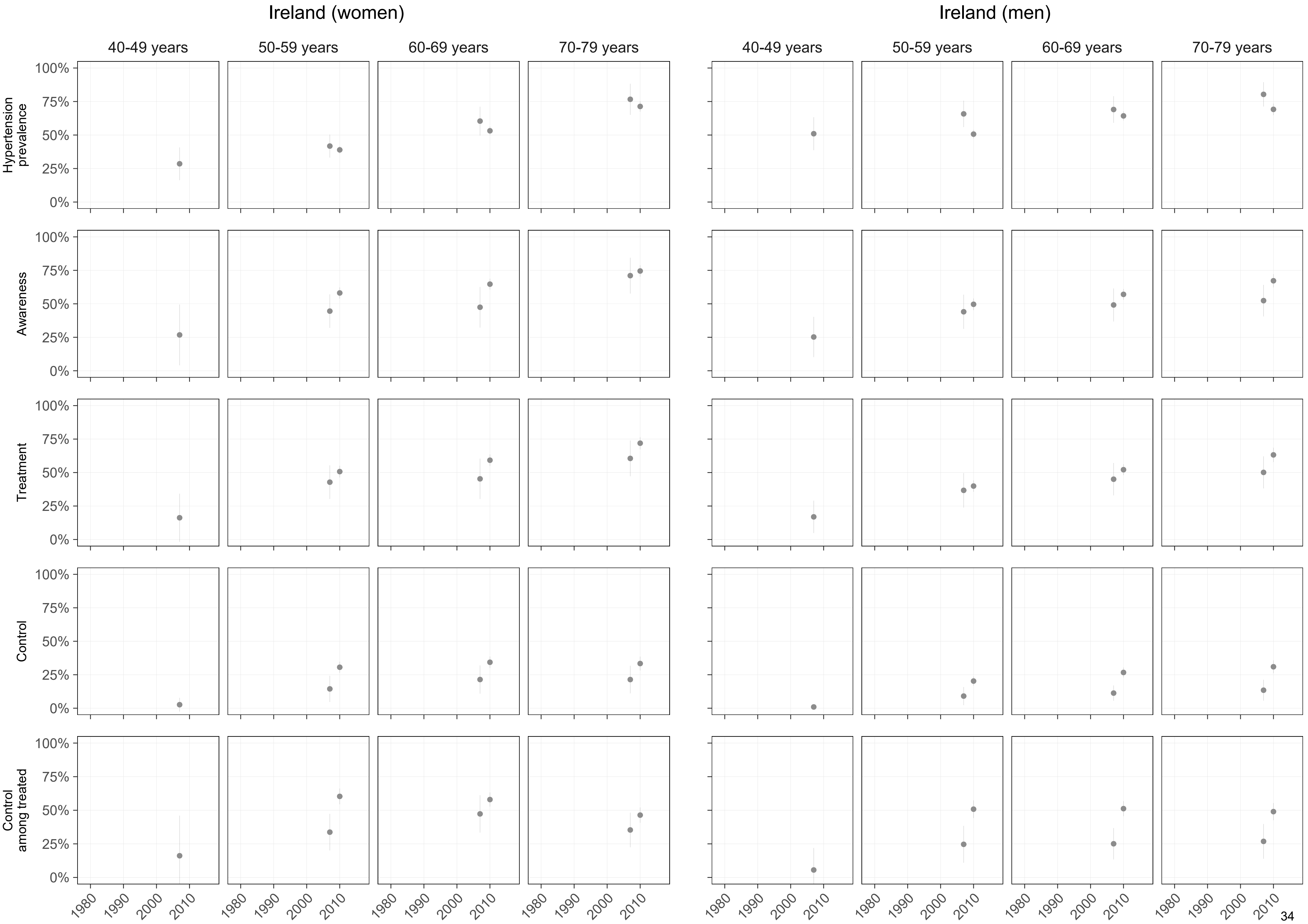
Ireland (women)
Ireland (men)
40-49 years
50-59 years
60-69 years
70-79 years
Hypertension prevalence
Awareness
Treatment
Control
Control among treated
0%
25%
50%
75%
100%
1980
1990
2000
2010

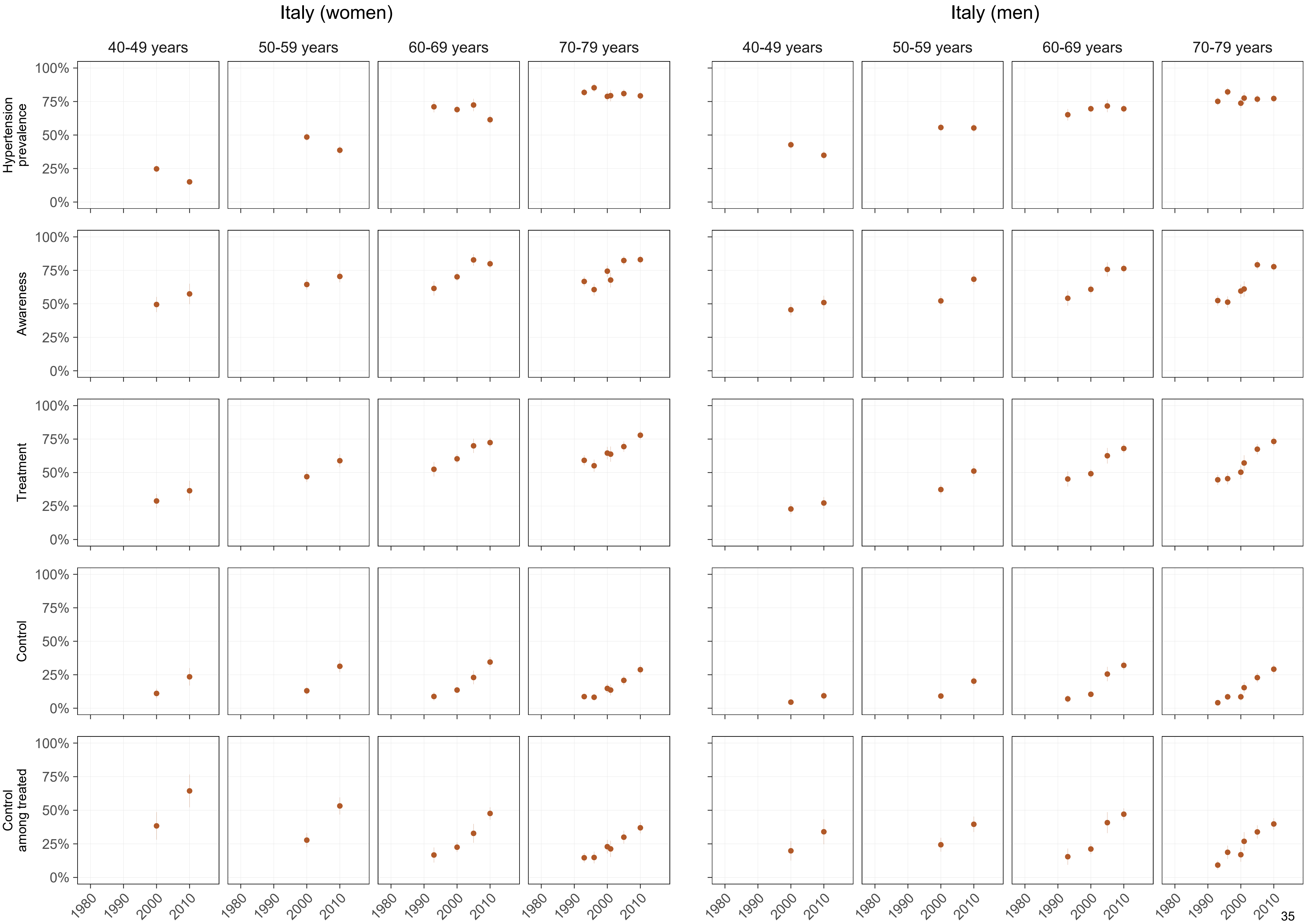
Italy (women)
Italy (men)
40-49 years
50-59 years
60-69 years
70-79 years
Hypertension prevalence
Awareness
Treatment
Control
Control among treated
0%
25%
50%
75%
100%
1980
1990
2000
2010

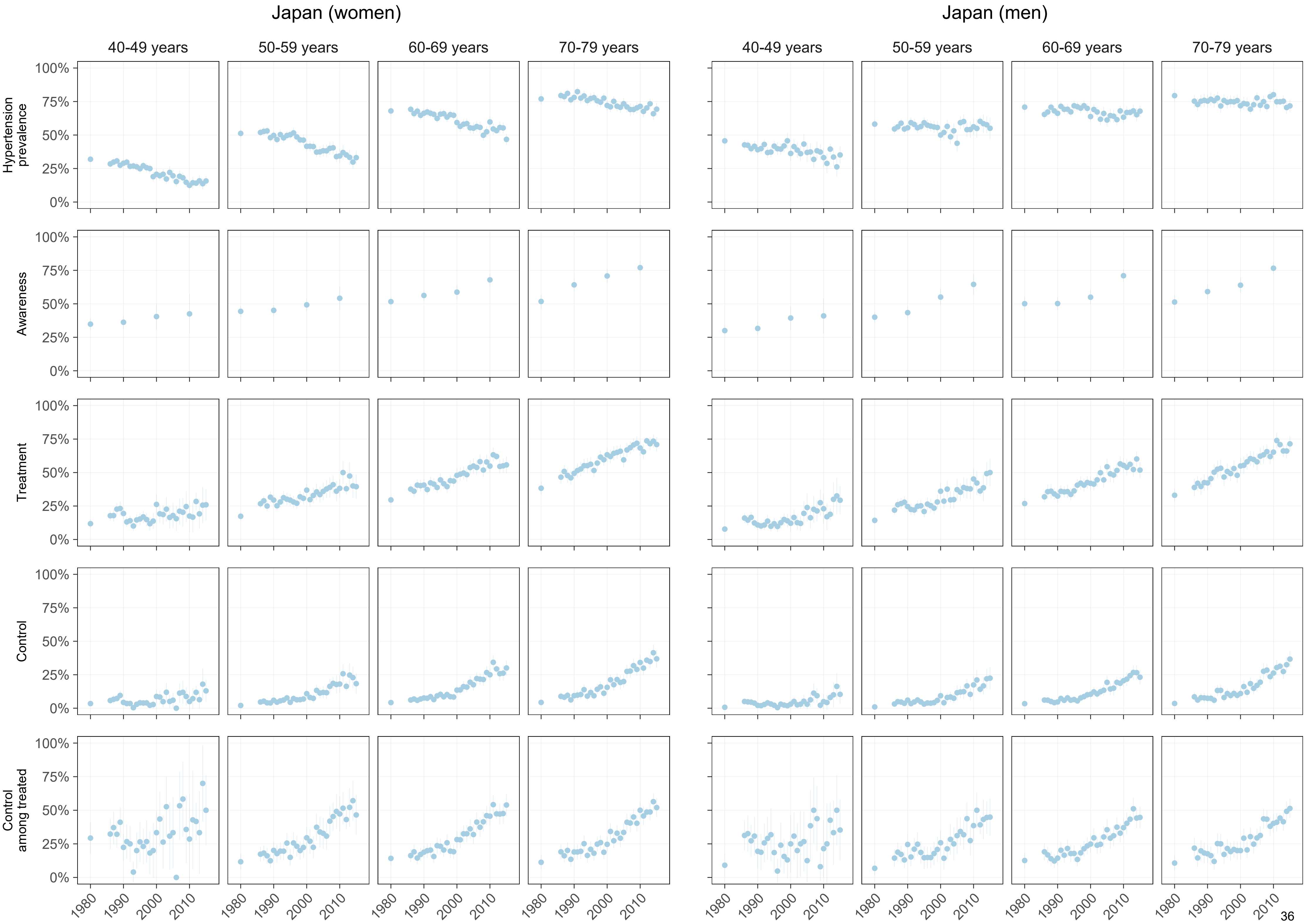

Japan (women)
Japan (men)
40-49 years
50-59 years
60-69 years
70-79 years
Hypertension prevalence
Awareness
Treatment
Control
Control among treated
100%
75%
50%
25%
0%
1980
1990
2000
2010

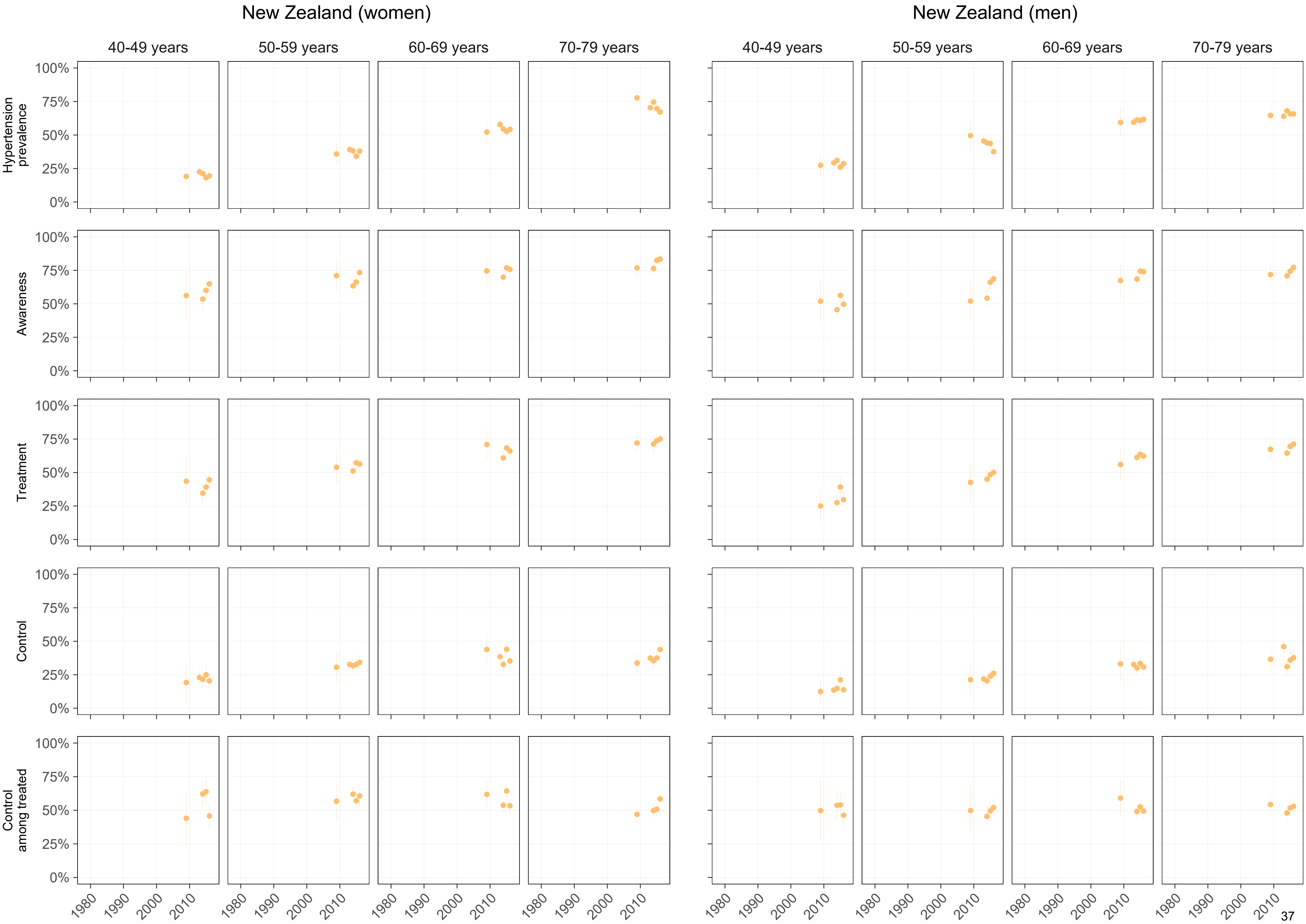

New Zealand (women)
New Zealand (men)
40-49 years
50-59 years
60-69 years
70-79 years
Hypertension prevalence
Awareness
Treatment
Control
Control among treated
0%
25%
50%
75%
100%
1980
1990
2000
2010

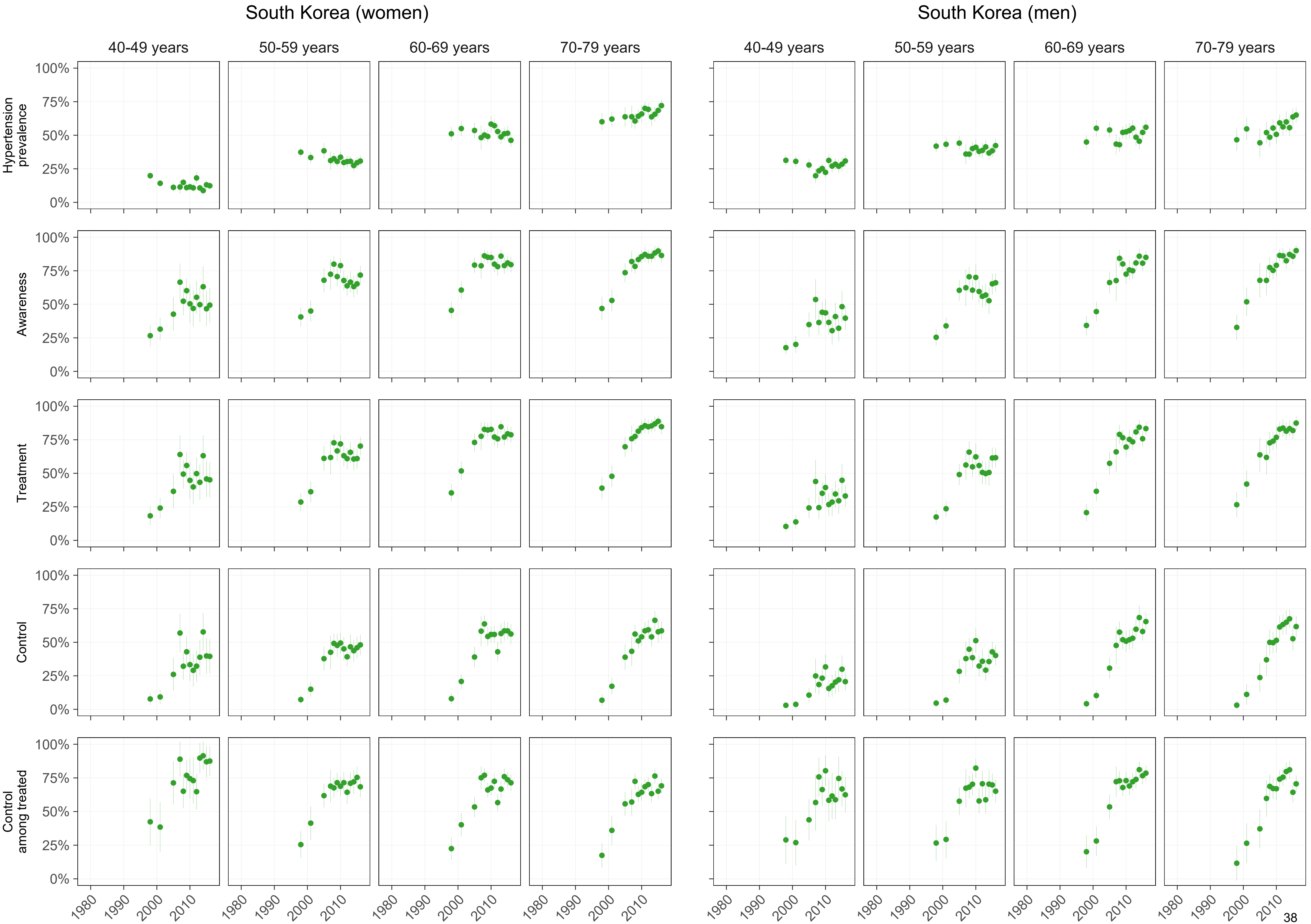

South Korea (women)
South Korea (men)
40-49 years
50-59 years
60-69 years
70-79 years
Hypertension prevalence
Awareness
Treatment
Control
Control among treated
0%
25%
50%
75%
100%
1980
1990
2000
2010

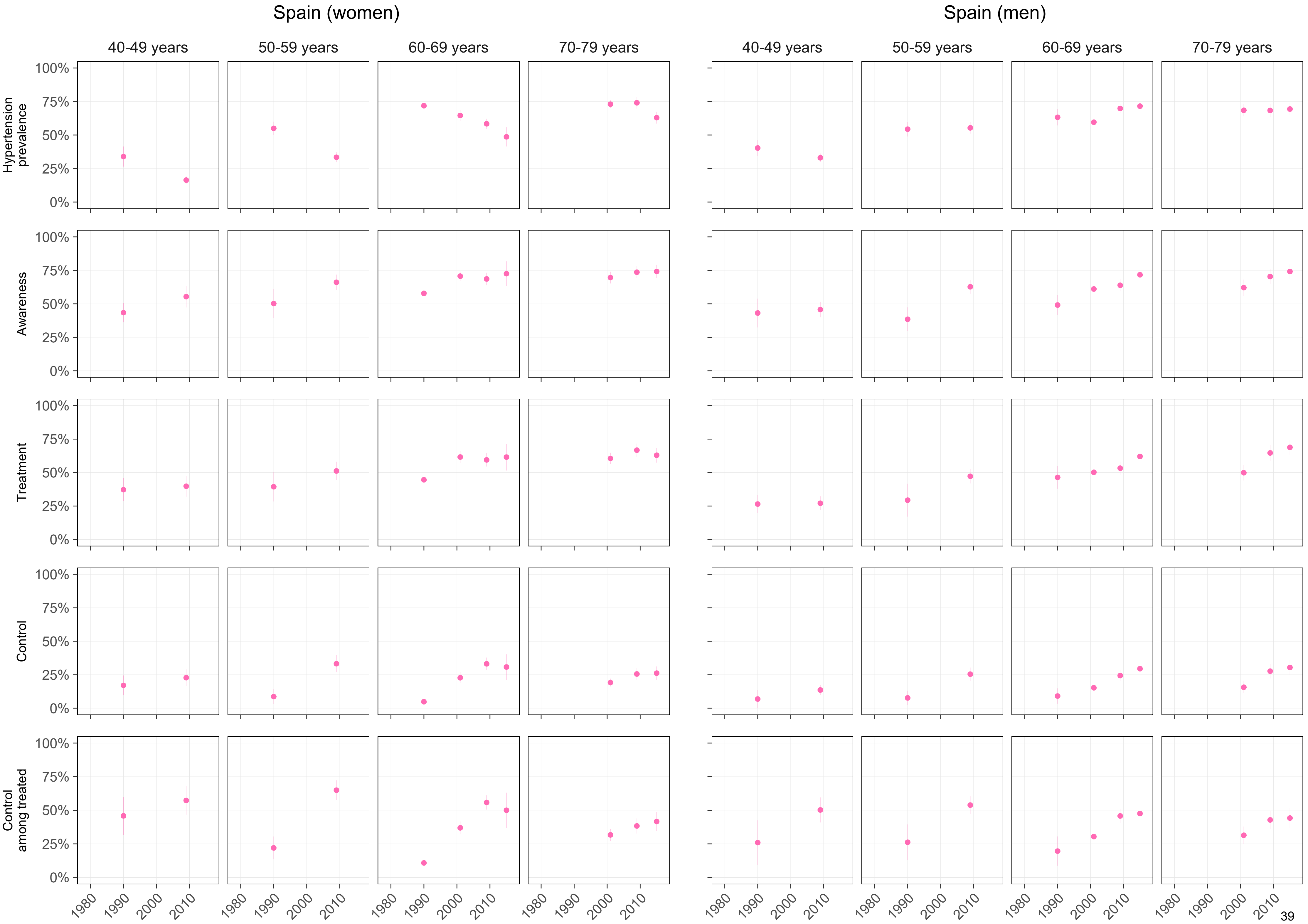
Spain (women)
Spain (men)
40-49 years
50-59 years
60-69 years
70-79 years
Hypertension prevalence
Awareness
Treatment
Control
Control among treated
100%
75%
50%
25%
0%
1980
1990
2000
2010

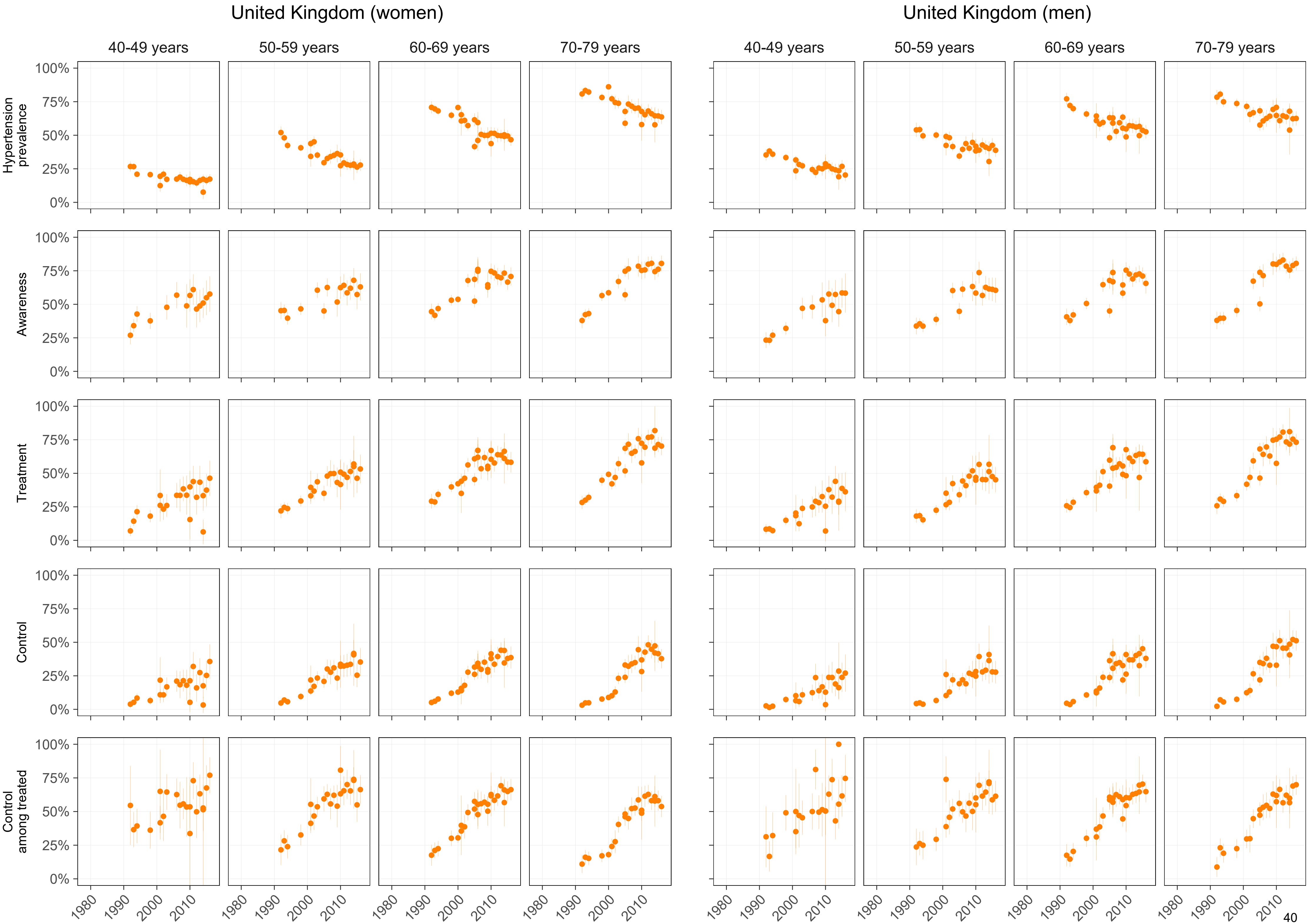

United Kingdom (women)
United Kingdom (men)
40-49 years
50-59 years
60-69 years
70-79 years
Hypertension prevalence
Awareness
Treatment
Control
Control among treated
0%
25%
50%
75%
100%
1980
1990
2000
2010

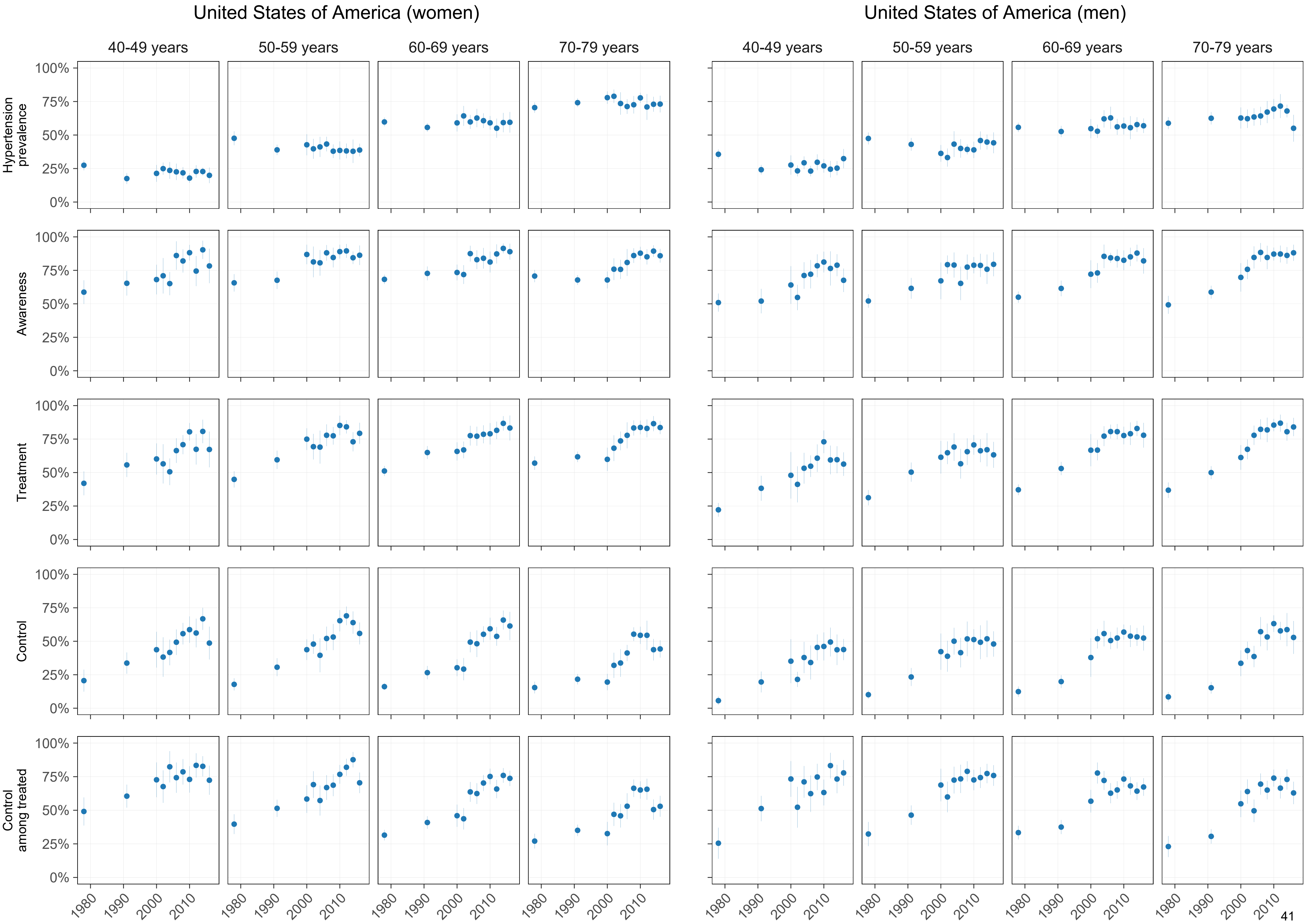
United States of America (women)
United States of America (men)
40-49 years
50-59 years
60-69 years
70-79 years
Hypertension prevalence
Awareness
Treatment
Control
Control among treated
0%
25%
50%
75%
100%
1980
1990
2000
2010

Appendix Figure 2. Distribution of participants by hypertension awareness, treatment, and control, and blood pressure status, in the latest national survey. Results shown are crude (ie, not age-standardised) to reflect the total burden of hypertension and its awareness, treatment, and control.

* Hypertension awareness rate for men with systolic blood pressure ≥160 mm Hg or diastolic blood pressure ≥100 mm Hg was unavailable due to Canadian regulations on releasing data on outcomes with small numbers.

† The latest national survey in Spain had data for 60 to 79 years; data from an earlier survey in 2009 were used for 40 to 59 years.

ǂ The latest national survey in Ireland had data for 50 to 79 years; data from an earlier survey in 2007 were used for 40 to 49 years.

§ The question on awareness was not asked in 2015 in Japan; awareness data from 2010 were used.

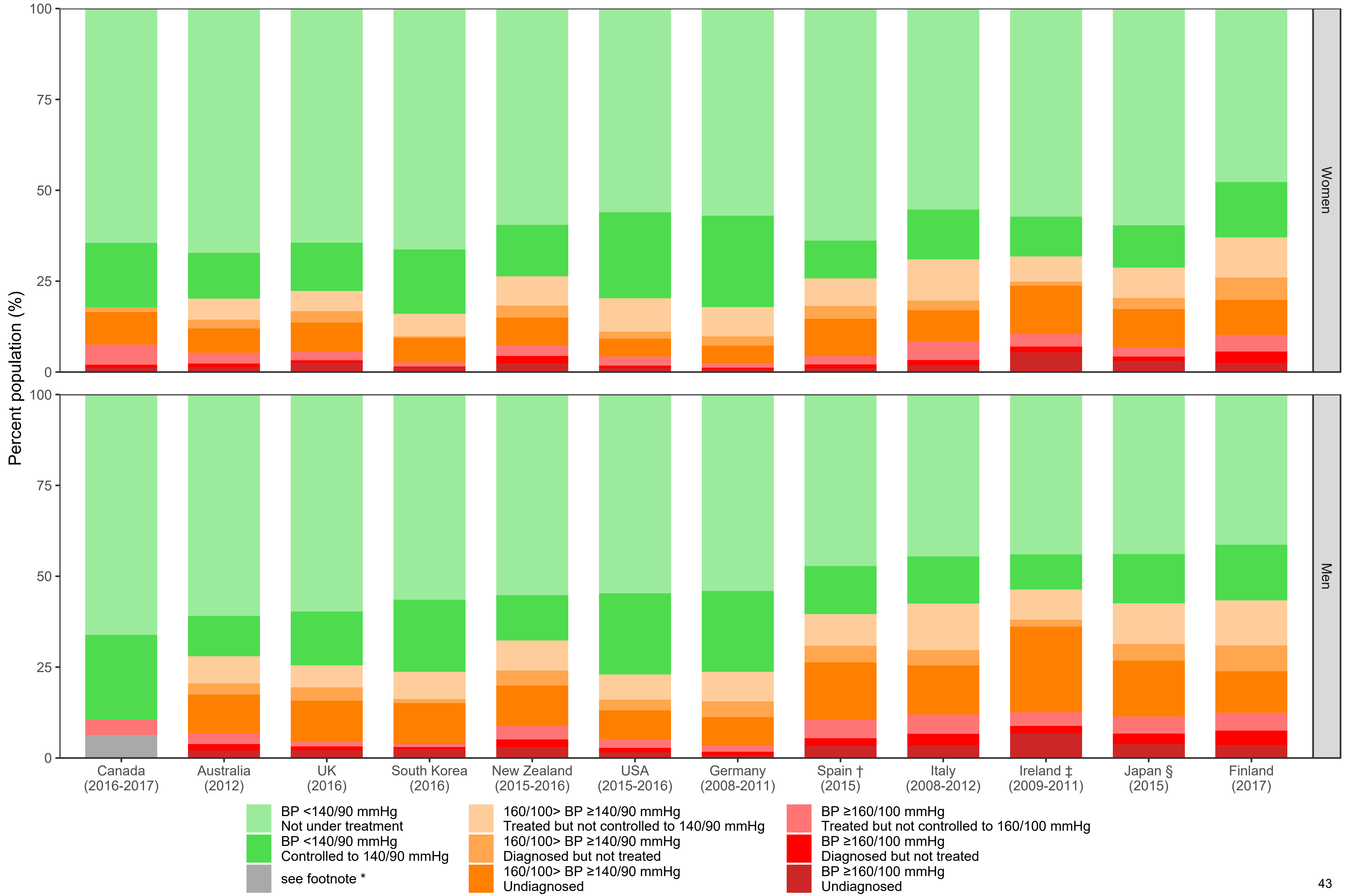
Percent population (%)
100
75
50
25
0
Women
Men
Canada (2016-2017)
Australia (2012)
UK (2016)
South Korea (2016)
New Zealand (2015-2016)
USA (2015-2016)
Germany (2008-2011)
Spain † (2015)
Italy (2008-2012)
Ireland ‡ (2009-2011)
Japan § (2015)
Finland (2017)
BP <140/90 mmHg Not under treatment
BP <140/90 mmHg Controlled to 140/90 mmHg
see footnote *
160/100> BP ≥140/90 mmHg Treated but not controlled to 140/90 mmHg
160/100> BP ≥140/90 mmHg Diagnosed but not treated
160/100> BP ≥140/90 mmHg Undiagnosed
BP ≥160/100 mmHg Treated but not controlled to 160/100 mmHg
BP ≥160/100 mmHg Diagnosed but not treated
BP ≥160/100 mmHg Undiagnosed
